# Influence of UV Irradiation Source and of Carboxylic Acid Additives on the Kinetics of Photodegradation of Five Tricyclic Antidepressant Drugs in Aqueous Medium

**DOI:** 10.3390/ijms27208940

**Published:** 2026-10-09

**Authors:** Maria D. Ciuca, Radu C. Racovita

**Affiliations:** Department of Inorganic Chemistry, Physical Chemistry and Electrochemistry, Faculty of Chemical Engineering and Biotechnologies, National University of Science and Technology POLITEHNICA Bucharest, 1-7 Gh. Polizu St., District 1, 011061 Bucharest, Romania; maria980804@gmail.com

**Keywords:** contaminants of emerging concern, pharmaceuticals, tricyclic antidepressants, gas chromatography–mass spectrometry, ultraviolet spectroscopy, UV photolysis, UV irradiation wavelength, carboxylic acids, rate constant, photodegradation time

## Abstract

Use of antidepressant medication has increased substantially nowadays. Investigations of their photostability in aqueous environment are therefore needed both for shelf life assessment of liquid pharmaceutical formulations and for development of wastewater remediation technologies. In this work, the comparative kinetics of photodegradation of five tricyclic antidepressants (TCAs) in aqueous solution was studied under direct photolysis using UVA, UVB, and UVC sources, respectively. A gas chromatography–mass spectrometry (GC-MS) method was developed and validated intra-laboratory for accurate quantification of the five TCAs (amitriptyline hydrochloride AMI, doxepin hydrochloride DOX, imipramine hydrochloride IMI, clomipramine hydrochloride CLO, trimipramine maleate TRI) in the course of photodegradation. UVC photolysis yielded the highest rates of photodegradation, followed by UVB and then UVA. The molecular structure and UV absorption characteristics of individual TCAs were shown to influence photodegradation rates as well. Specifically, heteroatoms (N, O, Cl) present in the molecule and redshifted UV absorption bands increased photosensitivity typically in the order: AMI < IMI < TRI < CLO < DOX. The effect that some mono- and polycarboxylic acids dissolved in the aqueous solutions had on TCA photodegradation kinetics under UVC was also investigated and, for most TCAs, rates were faster and durations required for their complete photodegradation decreased generally in the order: citric > oxalic > maleic > benzoic acid. Furthermore, citric acid was reused for a second round of TCA photodegradation and showed rate enhancement effects very close to those exhibited in the first round.

## 1. Introduction

Depression is the most common medical condition related to mental health in the 21st century, affecting people across cultures, age groups, and social backgrounds. The World Health Organization (WHO) estimated in 2022 that approximately 280 million people worldwide were living with some form of depressive disorder [1]. As awareness, diagnosis, and access to mental-health care have expanded, the prescription and consumption of antidepressant drugs have also increased: across 30 OECD countries, antidepressant consumption rose on average by 33% between 2010 and 2020 [2], while in high-income countries the increase was even greater [3,4]. Forecasts for 2030 present a continuing projected increase as generation Z showed higher levels of depression in the post-COVID pandemic years compared to older generations [5].

Antidepressant drugs include selective serotonin reuptake inhibitors (SSRIs), such as sertraline and fluoxetine; serotonin–noradrenaline reuptake inhibitors (SNRIs), such as venlafaxine and duloxetine; tricyclic antidepressants (TCAs), such as amitriptyline and imipramine; and less commonly used medicines such as monoamine oxidase inhibitors (MAOIs) or atypical antidepressants like trazodone and mianserin [4,6]. Unlike other classes whose name is related to the drug’s mechanism of action, TCAs owe their name to their molecular structures of either dihydrodibenzazepine or dihydrodibenzoheptafulvene, both of which incorporate three rings [7]. Although considered an older generation of antidepressants, TCAs are still prescribed nowadays in major depressive disorders and for patients whose depression symptoms have not improved with other newer-generation antidepressants [8]. Beyond depression, some TCAs can serve as augmenting drugs and sometimes even a first-line treatment for neuropathic pain, migraine, fibromyalgia, insomnia, pelvic pain, gastrointestinal syndromes, and psychiatric conditions different from depression [9].

Photodegradation is a key factor affecting the stability, efficacy, and safety of pharmaceutical formulations and can occur during manufacture, storage, and over the period of administration to the patient [10]. Therefore, pharmaceutical companies have long given ample consideration to the assessment of photostability of their products [11]. In addition, with their continuously increasing presence in society, antidepressant drugs and their metabolites have become environmental contaminants of emerging concern (CECs) [4], ending up in rivers, groundwater, and other water systems mainly through wastewaters, but also landfill leachate, pharmaceutical industry discharges, or improper disposal of expired medication [12]. Consequently, several methods have been developed and are still actively being sought for the removal of such CECs from wastewaters and other aqueous media. Some of the most common ones include adsorption on activated carbon or diosmectite [13], biological degradation using activated sludge [14] or fungal treatment [15], membrane treatment [16], ozonation [17] and several other advanced oxidation processes (AOPs) [18,19,20].

Direct UV photolysis has also been employed with greater or lesser effectiveness for removal of antidepressants from water and its applicability appears to be compound-specific [4]. By employing direct UVC photolysis (with emission centered at 254 nm) of six common antidepressants, Lin et al. obtained photolysis kinetic curves consistent with first-order reaction kinetics, with rate constants in the range 0.1–0.01 min^−1^ decreasing in the order: fluoxetine > paroxetine > sertraline > citalopram > fluvoxamine > venlafaxine [21]. These translated into degradation yields from as high as 90% within 30 min for fluoxetine to as low as 70% within two hours for venlafaxine [21]. Using also UVC photolysis, Nassar et al. managed to attain photodegradation yields of amitriptyline and clomipramine of 88 and 100% after seven hours of irradiation, while, in an AOP combining UVC with H_2_O_2_, photodegradation occurred faster [22]. The study by Khaleel et al. reached up to 92% trimipramine photodegradation after 128 min of irradiation using a UV-Vis source with several emission maxima between 200 and 600 nm [23]. Gros et al. studied the kinetics of photodegradation of desipramine over a period of three hours of irradiation using a solar simulator system with emission only above 290 nm (UVB + UVA + Vis) and determined a half-life of 36 h, while a related antipsychotic drug, amisulpride, photodegraded 12 times faster under the same conditions [24]. Maślanka et al. investigated the photodegradation for 114 days under a UVA source with a 365 nm emission maximum of antidepressants doxepin and venlafaxine, along with several other antipsychotic and sedative drugs, in both pure powder form and in powdered pharmaceutical formulation [25]. They observed a very different behavior of the two antidepressants, where 29% of doxepin and 39% of venlafaxine photodegraded as pure solids, and 72% of doxepin and 4% of venlafaxine in pharmaceutical preparations, suggesting that the nature of added excipients also influences photodegradation rates. For fluoxetine, Mendez-Arriaga et al. found a photodegradation yield of only 15% in basic aqueous medium with direct photolysis using UVA (centered around 360 nm) after 60 min, while AOPs combining UVA with O_3_ and peroxone (O_3_ + H_2_O_2_) attained yields above 97% in the same timeframe [19].

All these past studies suggest a variability in antidepressant photodegradation effectiveness using direct UV photolysis with several factors: the nature of the antidepressant, the specific UV range of the source, and additional substances present in the reaction mixture. In the work herein, we report an investigation of the kinetics of photodegradation of five different TCAs (Figure 1) in aqueous solution as a function of UV irradiation sources of exposure, spanning UVA, UVB, and UVC. For quantifying the concentrations of TCAs over the course of UV photodegradation, a gas chromatography–mass spectrometry (GC-MS) was developed and validated intra-laboratory. The kinetic findings were correlated with structural features and the UV absorption properties of the five TCAs. In addition, results regarding the potential improvement in TCA photodegradation effectiveness by four carboxylic acid additives are also reported.

## 2. Results and Discussion

### 2.1. Gas Chromatography–Mass Spectrometry (GC-MS) Method Validation

To ensure the accuracy of the quantification of tricyclic antidepressants (TCAs) in the aqueous samples subjected to photodegradation under various conditions, the GC-MS analytical method developed was validated intra-laboratory. For this purpose, calibration curves were constructed for each of the five TCAs, with incorporation of an internal standard (IS) at a concentration level of 1 μg/mL in all samples. Mirtazapine (MIR), a structurally related, albeit tetracyclic, antidepressant, was employed as IS in all chromatographic analyses to eliminate any possible errors caused by potentially unequal injection volumes [26]. Figures of merit of the GC-MS method and other relevant analyte characteristics are reported in Table 1, including the characteristic ion monitored (*m*/*z*) for each TCA, retention time (RT), relative response factor (RRF) with respect to the IS, linear range, R^2^ value for each calibration curve along its linear range, sensitivity (S) obtained from each analyte calibration curve, limit of detection (LOD) and limit of quantification (LOQ), percent recovery of TCAs from aqueous solutions evaporated to dryness followed by redissolution into chloroform, and relative standard deviation (RSD_R_) testing the reproducibility of recovery experiments.

RTs under working GC conditions were quite close for four of the five TCAs (17.2–17.5 min), likely due to the structural similarity of their molecules, but selected ion monitoring (SIM) operating mode allowed for clear discrimination and accurate quantification of individual analytes. RRFs with respect to IS (i.e., MIR, having a characteristic *m*/*z* of 195 and an RT of 17.8 min) were calculated using the equation:

(1) $${\mathrm{RRF}}_{\mathrm{TCA}}=\frac{A_{\mathrm{TCA}}}{C_{\mathrm{TCA}}}\cdot \frac{C_{\mathrm{IS}}}{A_{\mathrm{IS}}}\cdot \frac{M_{\mathrm{TCA}}}{M_{\mathrm{IS}}}$$

where *A_TCA_* and *A_IS_* are the peak areas (in arbitrary units) of the analyte TCA and IS, *C_TCA_* and *C_IS_* are the concentrations (in μg/mL) of analyte TCA and IS, and *M_TCA_* and *M*_IS_ are the molar weights (in g/mol) of analyte TCA and IS, respectively. RRFs were subunitary for four of the five TCAs, the only exception being TRI showing an RRF greater than one.

Linear ranges, supported by R^2^ values well above 0.99, were fairly wide, spanning four orders of magnitude for all analyte concentrations. Sensitivities (Ss), calculated as the slopes of calibration curves’ peak areas vs. analyte concentration, were in the order of 10^5^ to 10^6^ mL/μg. Limits of detection (LOD) and of quantification (LOQ) for each analyte were quite low, in the order of a few ng/mL, and were measured by injecting 20 blanks and assessing the standard deviation in peak areas using the characteristic *m*/*z* value at the expected retention time [27]. The equations used to calculate LOD_TCA_ and LOQ_TCA_ for an analyte TCA are given below:

LOD_TCA_ = 3∙σ_TCA_/S_TCA_ (2)

LOQ_TCA_ = 10∙σ_TCA_/S_TCA_ (3)

where σ_TCA_ and S_TCA_ are the standard deviation and sensitivity, respectively, for the analyte TCA.

Average recoveries from evaporated aqueous solutions of all five TCAs at both concentration levels tested (1 and 20 μg/mL) were in the range 97.2–102.0%, and showed good reproducibility, with RSD_R_ values in the range 2.04–3.91%, which are acceptable ranges for internal validation of analytical methods [28].

Because a part of our planned experimental studies was to also investigate the effect of four carboxylic acid additives (benzoic acid (BA), oxalic acid (OA), maleic acid (MA), and citric acid (CA)) on the photodegradation rates of the five TCAs in aqueous environment, we performed additional TCA recovery tests from aqueous solutions containing mixtures of a carboxylic acid with the same concentration as in photodegradation experiments and a TCA with each of the two concentrations mentioned above (1 and 20 μg/mL). The results are shown in Table S1. Average recoveries were in the range 94.8–101.6% with RSD_R_ values between 1.85 and 4.87% suggesting the additives do not significantly influence the quantitative redissolution of TCAs into chloroform after water evaporation.

### 2.2. Kinetics of Photodegradation of TCAs in Aqueous Solution Under Different UV Irradiation Wavelengths

The kinetics of photodegradation of individual TCAs in aqueous solutions was investigated using a photoreactor equipped with eight identical UV lamps. Three different sets of lamps were used, with different emission wavelengths, namely UVA, UVB, and UVC lamps, but also different emitted photon fluxes (see Section 3.4 below).

The initial concentration, *C*^0^*_TCA_*, of the aqueous solution of each TCA was set at 20.0 μg/mL. Samples were taken out of the photoreactor after various photodegradation durations, evaporated to dryness and redissolved in chloroform along with a fixed amount of IS, then analyzed chromatographically. The concentration, C*_TCA_*, of remaining (unreacted) TCA at a given time, in each sample subjected to photodegradation, was calculated using the RRFs reported in Table 1 against the known concentration of IS, *C_IS_*, using the formula:

(4) $$C_{\mathrm{TCA}}=\frac{C_{\mathrm{IS}}}{\mathrm{RR}F_{\mathrm{TCA}}}\cdot \frac{A_{\mathrm{TCA}}}{A_{\mathrm{IS}}}\cdot \frac{M_{\mathrm{TCA}}}{M_{\mathrm{IS}}}$$

where *RRF_TCA_* is the relative response factor of the particular TCA with respect to the IS, *A_TCA_* and *A_IS_* are the peak areas (in arbitrary units) of remaining TCA and IS in the chromatogram of each analyzed sample, *C_TCA_* and *C_IS_* are their concentrations (in μg/mL), and *M*_TCA_ and *M*_IS_ their molar weights (in g/mol).

For all TCAs, with the aforementioned initial concentrations and UV irradiation conditions, the experimental data could be fitted satisfactorily to pseudo-first-order reaction kinetics. This was not unexpected, as low concentrations of antidepressants, up to a few tens of μg/mL, as typically encountered in environmental samples, have been shown before to follow pseudo-first-order kinetics upon photodegradation [29,30,31,32,33,34,35]. At such low concentration levels, the antidepressant is the limiting factor and not the radiation received from the UV lamps, as explained previously in the literature [29]. Nevertheless, the numerical values of the apparent (observed) pseudo-first-order rate constants are affected by and incorporate within them contributions from factors such as the exact photon flux incident on the samples, the fraction of incident radiation absorbed by the sample, and the wavelength-dependent quantum yield. In the present work, we did not discriminate these components, hence the apparent rate constants reported hereon must be interpreted taking into account this limitation.

Apparent pseudo-first-order rate constants, *k*_1_, of photodegradation reactions could be evaluated for each TCA under each type of UV source according to the integrated first-order rate law (5):

(5) $$k_{1}=\frac{1}{t}\cdot \ln\left(\frac{C_{\mathrm{TCA}}^{0}}{C_{\mathrm{TCA}}}\right)$$

where *t* is the duration of TCA photodegradation (exposure time to UV light, in min), and *C^0^_TCA_* and *C_TCA_* are the initial and unreacted TCA concentrations at this time (in μg/mL), respectively.

The yield of photodegradation, *η* (%), could also be calculated for every photodegradation time *t*, according to Equation (6):

(6) $$\eta =\frac{C_{\mathrm{TCA}}^{0}-C_{\mathrm{TCA}}}{C_{\mathrm{TCA}}^{0}}\cdot 100$$

By combining Equations (5) and (6), a convenient Equation (7) is obtained, allowing the direct calculation of apparent rate constants *k*_1_ from photodegradation yields *η* attained after various photodegradation durations *t*:

(7) $$k_{1}=\frac{1}{t}\cdot \ln\left(\frac{100}{100-\eta }\right)$$

If pseudo-first-order kinetics does indeed describe the observed photodegradation processes, then, according to Equation (7), plots of ln[100/(100 − *η*)] as a function of *t* will be linear and their slopes will be equal to the apparent rate constants *k*_1_ of the respective TCAs under the corresponding UV irradiation source.

Figure 2 shows the kinetic curves obtained for the photodegradations of the five TCAs upon exposure to UVA, UVB, and UVC sources, respectively. In all instances, linear fits were excellent, with R^2^ values of 0.98 or higher. This proves that all TCA photodegradation reactions in aqueous medium follow pseudo-first-order kinetics under the UV radiation wavelengths and fluxes used, and considering the concentration levels employed (≤20 μg/mL).

Figures S1–S5 in the Supplementary Materials exemplify relevant portions of the SIM chromatograms corresponding to AMI, DOX, IMI, CLO, and TRI, in this order, at various time-points in the course of their photodegradation using the UVC source. As UVC exposure time increases, it can easily be noticed in all these figures that the peak area of the respective TCA diminishes in comparison to that of the co-injected internal standard with constant concentration.

Dark control experiments were also performed to ensure that the observed TCA concentration decays in time were indeed due to UV light exposure and not other causes like thermal degradation, volatilization, hydrolysis, adsorption onto glassware, or handling-related losses. These control experiments were performed for the same duration as the longest UV exposure time applied above for each TCA, using both TCA-only aqueous solutions and aqueous solutions containing mixtures of TCA and a carboxylic acid (the latter in relation to Section 2.4 below). The timecourses of TCA concentrations in the solutions kept in the dark are included in Table S2, where they are compared against the TCA concentrations measured during the UV photodegradation experiments, i.e., the concentrations that were used to generate the kinetic plots shown in Figure 2. All solutions kept in the dark maintained their initial concentrations of TCAs, within experimental error limits, indicating that all TCAs are stable in water in the absence of UV irradiation for several hours, both without and with a co-existing carboxylic acid in their solution.

The apparent pseudo-first-order rate constants, *k*_1_, for all photodegradation processes, i.e., the slopes of the kinetic curves shown in Figure 2, are reported in Table 2. The same table also reports calculated UV exposure durations needed for complete photodegradation of each TCA under each type of UV source, denoted *t*_∞_. These were evaluated considering in Equation (7) a photodegradation yield *η* equals to 99.99% (practically complete photodegradation), which results in the following formula for calculation of *t*_∞_:

(8) $$t_{\infty }=\frac{\ln10000}{k_{1}}$$

Several trends can be observed from the data in Table 2.

First, as the UV irradiation source is switched from UVA to UVB and then UVC, the rate of photodegradation increases for practically all TCAs, hence the photodegradation time required for their complete photodegradation, *t*_∞_, gets shorter. While under UVA irradiation it can take up to 10,233.7 min (around 7 days) to completely photodegrade some of the TCAs. UVB reduces *t*_∞_ to at most 2141.9 min (about 1.5 days) and UVC to no more than 220.3 min (less than 4 h). This influence of UV irradiation source can be interpreted to some extent. As the emission maxima of the UV sources shift towards lower values, the energy of photons increases and hence UVC irradiation is more aggressive than UVB, which in turn is more aggressive than UVA [36]. The UVC lamps used also had the highest total photon flux during the experiments, but the total flux of UVB lamps was considerably lower than that of UVA lamps (see Section 3.4), yet all TCAs photodegraded much faster with UVB. Hence, the wavelength of projected UV radiation is a factor of influence on photodegradation kinetics in addition to photon flux. Furthermore, organic aromatic molecules like the TCAs herein typically have more absorption bands toward lower wavelengths in the UV domains; thus absorption of UVC by TCA molecules will be more efficient than of UVB and absorption of UVB will be higher than that of UVA (see Section 2.3 below).

Second, there is a strong dependence of photodegradation rates and completion durations on the nature of TCAs. The fastest photodegradation is observed in the case of DOX at all wavelengths, having a *t*_∞_ ranging from 397.0 min (under 7 h) with UVA to only about 12 min with either UVB or UVC. DOX is seconded by CLO, with *t*_∞_ ranging from 1334.8 min (just over 22 h) under UVA irradiation to around 40 min when using UVB or UVC. Third is TRI with a *t*_∞_ under UVA of 2631.5 min (just under 44 h) going down to 351.5 min (nearly 6 h) under UVB and further down to 94.5 min (about 1.5 h) under UVC irradiation. Slowest to photodegrade are AMI and IMI. With UVA, AMI and IMI exhibit the longest *t*_∞_ around 9000–10,000 min (approx. 7 days). Using UVB, IMI degrades completely in 736.8 min (around 12 h), which is about three times faster than AMI with 2141.9 min (around 36 h). Similarly, with UVC, IMI undergoes complete photodegradation in 153.3 min (around 2.5 h) while this takes 220.3 min (over 3.5 h) in the case of AMI.

From these observations it follows that the molecular structures of TCAs (Figure 1) influence their ease of photodegradation. The existence of a larger number of heteroatoms in the molecule, such as the additional O in DOX and the Cl in CLO, is clearly a factor that decreases photostability under UV exposure, while molecules with fewer heteroatoms, like IMI and especially AMI, tend to be less prone to UV photodegradation. This difference in photostability is likely due to the increased likelihood of light-driven homolytic cleavage of bonds that involves such heteroatoms, i.e., C–O bonds and C–Cl bonds, leading to unstable radical intermediates that further degrade, which are photochemical phenomena well documented in the literature [37,38,39,40,41,42]. However, TRI also exhibited a faster rate of photodegradation than IMI, even though their structures share the exact same heteroatoms (Figure 1). In this case, one contributing factor may be the additional methyl branching of the aliphatic chain of TRI, which increases the probability of homolytic cleavage and radical formation at the location of branching where a tertiary carbon is present [43,44]. Another contributing factor is, however, the nature of the anion in the TRI salt we used in our aqueous photodegradation experiments (see Section 3.1). While the other four TCAs were employed in the form of water-soluble hydrochlorides, due to lack of a commercially available TRI hydrochloride of analytical grade, a TRI maleate salt was purchased and used instead. As shown below (Section 2.4), carboxylic acids, including maleic acid, when present in the same solution as TCAs, enhance the observed rate of UV photodegradation of the latter.

The past literature on kinetic aspects of direct UV photolysis of TCAs is scarce. One study reported pseudo-first-order rate constants for the UVC photodegradation of AMI and CLO in aqueous medium of 0.0399 min^−1^ and 0.3678 min^−1^ respectively [22], both of which are very close to our results. A recent communication assessed IMI photodegradation under direct UVC in 8 mmol/mL aqueous solution and concluded that 350 min was necessary to achieve 90% degradation yield [45], which was more than twice longer compared to our findings. Another study reported for AMI dissolved in tap water an apparent rate constant of 0.0366 h^−1^ (0.0006 min^−1^) under direct photolysis with a visible + near UV source [34], a value that was similar in magnitude to our result for AMI when using UVA. The direct photolysis of DOX was only attempted on the pure solid form with a UVA source and a mere 29% photodegradation yield was obtained after 114 days of irradiation [25]. This may appear a lot slower than what we found, but a comparison should not even be made, since photochemical reactions in liquid state occur with completely different kinetics than their solid state counterparts, where numerous additional factors come into play, such as diffusivities, defects, polymorphs, surfaces, particle size distribution, particle shape, pore size and distribution [46]. In a different study [23], photodegradation of aqueous solutions of TRI (also in the form of maleate salt) was undertaken using a broad spectrum UV source with nine emission maxima from as low as 256 nm to as high as 436 nm, hence spanning all of UVC, UVB, UVA, and even Vis, which makes it difficult to make direct comparisons with our data. Under these conditions, although their kinetic data could be fitted to the first order, the authors reported variable first-order rate constants between 0.0178 and 0.3249 min^−1^ depending on the initial concentrations of TRI, which ranged from 5 to 100 μg/mL [23]. This variability is inconsistent with first-order reactions, which the authors acknowledged themselves and attributed to a combination of mechanisms simultaneously at play leading to mixed reaction orders. In fact, the concentration of the antidepressant has been shown to have influence in some direct UV photolyses on the observed reaction order. For the tetracyclic mianserin, Wawryniuk et al. reported first-order photodegradation kinetics and a rate constant of 0.246 h^−1^ starting with 20 μg/mL of antidepressant in water and using full spectrum light (UV-Vis) [32], while Sfair et al. found zero-order kinetics with a rate constant of 0.279 μg mL^−1^ min^−1^ starting with 400 μg/mL in methanol and using exclusively UVC [47]. Trawinski and Skibinski provided an explanation for these apparently conflicting results [29]. Pseudo-first-order kinetics has been most commonly observed in direct photolysis of pharmaceuticals, but this was because most studies operate with low concentrations of the studied compound and thus low values of solution absorbance. Under such conditions, the photoreaction rate is directly proportional to substrate concentration in agreement with pseudo-first-order rate law. On the other hand, at a high concentration of substrate, radiation intensity becomes limiting and rates become independent of concentration, i.e., zero-order kinetics takes over [29]. In our present work, TCA concentrations were never that high to observe this switch in kinetic behavior.

### 2.3. Ultraviolet–Visible (UV-Vis) Absorbance Spectra of TCAs

The different kinetics of UV photodegradation of the five TCAs could also, in part, result from different UV absorption efficiencies of these molecules. Thus, UV-Vis absorbance spectra of the five TCAs in aqueous medium were measured in order to establish the UV absorption characteristics of each TCA, which could potentially be correlated with their different photodegradation behaviors (Figure 3).

From the recorded spectra, it is immediately apparent that all TCAs absorb most strongly in the UVC domain (below 280 nm [48,49]), which explains why the fastest rates of photodegradation were obtained with the UVC lamps in all cases (Table 2). For dihydrodibenzoheptafulvenes (AMI and DOX), two absorption maxima are observed in the UVC range of their spectra, one just over 200 nm and the second approaching 250 nm. On the other hand, dihydrodibenzazepines (IMI, CLO, and TRI) exhibit three local maxima in the UVC domain of their absorption spectra, the first two at approximately 210 nm and 250 nm, hence near to those of AMI and DOX, while the third one is the most redshifted to a wavelength just under 280 nm. The additional absorption band of dihydrodibenzazepines is a consequence of the additional N atom they incorporate in their tricyclic part, which induces n → π* transitions while also bathocromically shifting π → π* bands through extended conjugation [22].

Interestingly, in the UVB domain (280–315 nm [49,50]), DOX was the only antidepressant to show a local absorption maximum at about 295 nm. Most probably, the extra heteroatom, O, extends the conjugation of one aromatic ring and the exocyclic C=C bond causing a bathocromic shift in the π → π* band in comparison to AMI. This unique spectral feature of DOX explains why this TCA was the only one to exhibit nearly identical photodegradation rate with the UVB source as with the UVC source (Table 2). The aforementioned UVC absorption bands peaking around 280 nm of IMI, CLO, and TRI are tailing into the UVB domain (Figure 3), which also explains the higher rates of UVB photodegradation of these three TCAs as compared to AMI that lacks this spectral feature and is thus less photosensitive. It should be noted, however, that, according to manufacturer’s technical specifications, all our UV irradiation sources have emission spectra spread out over a range of wavelengths centered more or less around the maxima mentioned in Section 3.4. Thus, UVB lamps emit to some extent UVC and UVA radiation as well, meaning that AMI will eventually photodegrade under the UVB source, albeit slower than with UVC lamps. The same holds true for UVA lamps and explains why all TCAs ultimately also photodegrade under UVA exposure, but with the slowest rates (Table 2).

### 2.4. Influence of Carboxylic Acids on UVC Photodegradation Rates of TCAs

Because one of the TCAs was only commercially available as maleate salt and not hydrochloride as the other four, we investigated whether carboxylic acids added to the TCA aqueous solution have an influence on photodegradation rates of TCAs. Since UVC effected the most rapid photodegradation process, the influence of carboxylic additives was tested using this irradiation source. Moreover, the carboxylic acid functional group (COOH) does not absorb UV light above 260 nm [51], so, if an effect were to be observed, it would not likely be enabled by UVA or UVB sources.

Four carboxylic acids were employed as photodegradation additives: one monocarboxylic acid (benzoic acid (BA)), two dicarboxylic acids (oxalic acid (OA) and maleic acid (MA), respectively), and one tricarboxylic acid (citric acid (CA)). Figure 4 shows a comparison of complete photodegradation durations, *t*_∞_, attained for the five TCAs in the absence and presence of each of these additives.

From the results obtained (Figure 4) it can be concluded that carboxylic acid additives increase to some extent the photodegradation rates of TCAs and consequently lower more or less the photodegradation durations needed to reach complete photodegradation. For DOX, which already had a sufficiently short t_∞_ without any additive, there was no significant shortening of photodegradation time with either of the four carboxylic acids tested. All other TCAs were, however, affected by some or all of the additives. CA followed by OA were the additives that most significantly decreased t_∞_ for all four remaining TCAs, i.e., AMI, IMI, CLO, and TRI. MA also significantly shortened t_∞_ for AMI, IMI, and TRI, but no sizeable effect was observed for CLO. BA addition only impacted the t_∞_ of IMI and CLO, leaving AMI and TRI largely unaffected. In terms of magnitude of decrease in t_∞_ values, CA stands out once again, reducing IMI photodegradation time about six times, while for other TCAs no more than two times. OA follows, with, at best, a four-fold reduction in t_∞_ also attained in the case of IMI, while MA only allowed for a maximum three-fold decrease in the t_∞_ of IMI. BA caused the least significant reduction in t_∞_ in the two cases where it did, no better than two-fold even for IMI.

The possibility of recovery and reuse of the remaining carboxylic acid additive at the end of a photodegradation experiment for a new photodegradation round of a fresh batch of TCA was explored in the case of citric acid (CA), the additive that performed best. After two hours of continuous photodegradation of the first batch of each TCA, which is a duration sufficient for practically complete photodegradation in the presence of CA according to the data in Figure 4, the remaining CA after evaporation to dryness was redissolved in the same test tube using a fresh aqueous solution of the same TCA and photodegradation was repeated, with sampling after various periods of time for GC analysis in order to establish the *k*_1_ and t_∞_ of photodegradation of each TCA in the presence of recycled CA. The results obtained using recycled CA in comparison to fresh CA are shown in Table 3.

From the data in Table 3, it can be concluded that the reuse of the same CA in the second photodegradation round did not significantly alter the kinetics of photodegradation for four of the five TCAs in comparison to that observed in the first round using fresh CA, the only notable exception being TRI where a decrease in the apparent rate constant and, implicitly, an increase in the complete photodegradation time were observed. This suggests that most of the original CA added at the beginning of the first photodegradation round is recovered from the aqueous solution after two hours of UVC irradiation and can successfully serve as photodegradation additive for a second round. This conclusion is supported by past studies about the UV photostability of some polycarboxylic acids, which have shown citric acid, along with several dicarboxylic acids, to be retrieved largely unaffected after four hours of irradiation with a medium-pressure Hg lamp emitting all of UVA, UVB, and UVC combined [52].

The mechanism of photodegradation of TCAs under UVC light (Scheme 1) has been the object of study of several published articles [22,23,24,25,29,33,35,53,54,55,56,57,58]. The first step is photoexcitation of the TCA molecule upon absorption of UVC light yielding an excited singlet state (TCA*) that may undergo intersystem crossing to an excited triplet state (^3^TCA*). This can subsequently undergo direct photochemical fragmentation and/or photooxidation by sensitized oxygen. In the former case, hydrogen abstraction, dehydrogenation, and cleavage reactions turn ^3^TCA* into radical intermediates (TCA•) that further cleave or react with dissolved oxygen (O_2_) in the aqueous environment and generate peroxyl radicals (TCA-O-O•), which in turn undergo hydrogen abstraction, fragmentation, hydroxylation, and further oxidation to a variety of oxidized products dependent upon the exact nature of the TCA [56]. In the latter case, ^3^TCA* can transfer either energy to regular (triplet) dissolved O_2_ forming singlet oxygen (^1^O_2_) or even an electron forming the superoxide radical anion (O_2_•^−^), both as reactive oxygen species (ROS) that were identified in past studies [54,59,60]. Through protonation, the conjugate acid of the superoxide, i.e., the HO_2_• radical, may also arise, as well as H_2_O_2_ molecules and HO• radicals through subsequent coupling and dissociation reactions, respectively. All these ROS effect secondary oxidation of TCA molecules to an even wider variety of products, such as hydroxylation [22,23,25,55,56,57], hydration of alkene bonds [22,25,55], dehydrogenation [33], *N*-demethylation [23,25,33,55,56,57,58], and *N*-oxidation products [55,58]. Supplementary Table S3 includes a summary of structures of such intermediate oxidation products that have been reported in the literature. With prolonged exposure to UVC, these oxidized products go through multiple rounds of side chain fragmentation, ring cleavage, and further oxidation ultimately resulting in inorganic end products (e.g., CO_2_, H_2_O, NH_4_^+^, NO_2_^−^, NO_3_^−^, Cl^−^), a process known as mineralization [4,29,35].

The mechanism by which carboxylic acids assist the photodegradation of TCAs can only be speculated at this time. From the data obtained, kinetic effects on TCA photodegradation were observed for all types of carboxylic acids, whether classified as mono- (BA), di- (OA and MA), and tricarboxylic acids (CA), or as saturated (CA and OA), unsaturated (MA), and aromatic acids (BA). What they all have in common is the existence of at least one COOH in their molecules, so a general mechanism likely revolves around this functional group. In the simplest version, COOHs in the carboxylic acids (R-COOH) absorb part of the incoming UVC radiation turning first into singlet high-energy excited states (R-COOH*), which, through intersystem crossing, become triplet excited states (^3^R-COOH*) that transfer this excess energy to neighboring TCA molecules, which then become excited themselves (TCA*) and can undergo photodegradation following the same mechanism shown in Scheme 1 from the second step onwards. An analogous photosensitized degradation mechanism has been previously described in aqueous environment for AMI and its metabolite, nortryptiline, in the presence of fulvic acid [33], as well as for IMI in the presence of humic substances [57]. This alternative carboxylic acid-assisted mechanism is most likely happening synchronously with the regular one from Scheme 1, meaning that only a part of the TCA molecules will get excited via ^3^R-COOH*, while others will reach their excited state via direct UVC absorption. This is consistent with our finding that certain TCAs (especially DOX) did not improve their photodegradation kinetics upon addition of some of the carboxylic acids (Figure 4), probably because the regular photodegradation mechanism (Scheme 1) was itself faster than the assisted path in these particular cases.

Other more complicated paths could also be envisaged, where aggressive radicals, like carboxyl RCOO∙ and its decarboxylation product, alkyl R∙, are generated under UVC [42,61] and cause further degradation of TCAs. These paths are, however, less likely, because the COOH functionality is quite stable even under UVC, being often cited as the end product of many photodegradation processes in aqueous medium [62,63,64], while photodecarboxylation of carboxylic acids was shown to be made possible only by employing photocatalysts [65] or by direct photolysis with vacuum UV radiation [66].

Very few other studies investigating the effect of carboxylic acids as additives employed during the direct photolysis of pharmaceuticals have been published. One particular study on the photolysis of AMI in 10 μM aqueous solutions showed this drug was resistant under simulated sunlight (>300 nm) for 4 h, but, when 20 μg/mL fulvic acid (a high-molecular-weight humic substance) was added to the solution, photodegradation became significant and a pseudo-first-order rate constant of 0.24 h^−1^ (0.004 min^−1^) could be measured [33]. Several other reports exist about the enhancement by carboxylic acids of photocatalytic degradation kinetics of certain pesticides [67], pharmaceuticals [68], commodity organic chemicals [61,69,70], and inorganic pollutants [71] found in water. However, all these studies involved heterogeneous UV irradiation processes using solid photocatalysts like TiO_2_, CeO_2_, Fe_2_O_3_ or Fe_3_O_4_ and the rate enhancement was often attributed to an activation of the photocatalyst through carboxylic acid interactions with its surface or a metal–carboxylate complex formation rather than direct interactions of carboxylic compounds with the substrates subjected to photodegradation [61,67,68,69,70,71]. Of particular interest, given the nature of additives used, is a study that investigated the effect of six low-molecular-weight carboxylic acids on the heterogeneous photodegradation using ferrihydrite mineral as a photocatalyst of aqueous sertraline subjected to a xenon lamp emitting visible light, UVA, and UVB combined [68]. The authors found the rates of carboxylic acid-aided photodegradation to decrease in the order: citric acid > tartaric acid > malic acid > lactic acid > succinic acid > malonic acid, with a *t*_∞_ value of only 85 min in the case of citric acid and going up to nearly 9500 min in the case of malonic acid [68]. While citric acid outperformed the other carboxylic acids tested in their study just as it did in our work herein, there is little relevance to such an analogy considering the very different mechanism at play in this heterogeneous photocatalytic process.

## 3. Materials and Methods

### 3.1. Chemicals

All tricyclic antidepressants (TCAs), amitriptyline hydrochloride (AMI, ≥98%), doxepin hydrochloride (DOX, ≥98%, mixture of 85% E isomer and 15% Z isomer), imipramine hydrochloride (IMI, ≥99%), clomipramine hydrochloride (CLO, ≥98%), and trimipramine maleate (TRI, ≥98%), were purchased from Sigma Aldrich (Burlington, MA, USA). Mirtazapine (MIR, ≥98%) was from Thermo Fisher Scientific (Waltham, MA, USA). Benzoic acid (BA, ≥99.5%), oxalic acid dihydrate (OA, ≥98%), maleic acid (MA, ≥99%), citric acid monohydrate (CA, ≥99.5%), and chloroform (≥99.9%, with amylenes as stabilizer) were from Sigma Aldrich. Helium 6.0 (≥99.9999%) was purchased from Linde GmbH (Pullach, Germany). All reagents were used as received. Doubly distilled water produced with an in-house distillation system was used in all experiments.

### 3.2. Preparation of Solutions

Aqueous solutions with exact concentrations of each TCA were prepared by accurately weighing the necessary amount of TCA on a Kern ABD 200—4 analytical balance (KERN GmbH, Bensheim, Germany), with a precision of ±0.0001 g, and dissolving in the corresponding amount of water. Stock solutions were typically 200 μg/mL, except for TRI where the stock was 40 μg/mL due to its limited solubility in water. More dilute solutions were prepared by controlled dilution from the stock solutions.

The same procedure was applied for the preparation of TCA and MIR solutions in chloroform for the calibration of the GC-MS (gas chromatograph–mass spectrometer). With chloroform as the solvent, the preparation of stock solutions with a concentration of 200 μg/mL, i.e., higher than 40 μg/mL, was possible for all antidepressants, including TRI. Solutions with lower concentrations were prepared by serial dilutions from the stock using pure chloroform.

### 3.3. Gas Chromatography–Mass Spectrometry (GC-MS) Analyses

The gas chromatograph used was a Thermo Fisher Scientific Focus GC and was coupled to a Polaris Q ion trap mass spectrometer (Thermo Fisher Scientific). Automated injection was employed, using the interfaced TriPlus Autosampler (Thermo Fisher Scientific), in splitless mode, while maintaining the inlet at a temperature of 250 °C. The injection volume was always 1 μL. Separation was done on a capillary column TR-5MS (Thermo Fisher Scientific; length = 30 m; internal diameter = 0.25 mm; film thickness = 0.25 μm). Helium was employed as the mobile phase, at a constant flow rate of 1 mL/min. The ion source temperature was 250 °C, and the MS transfer line was kept at 280 °C. The GC oven was programmed as follows: start at 60 °C; hold for 3 min; raise to 300 °C at 15 °C/min; hold for 4 min [72]. All quantification experiments were performed using the selected ion monitoring (SIM) mode, in order to enhance sensitivity, similar to other published studies [72,73,74,75,76,77]. The ions chosen for quantifying each TCA and the internal standard were: *m*/*z* of 58 for AMI; *m*/*z* of 58 for DOX; *m*/*z* of 234 for IMI; *m*/*z* of 268 for CLO; *m*/*z* of 249 for TRI; and *m*/*z* of 195 for MIR. All of these product ions were selected because they produced the most intense peaks in the electron ionization mass spectra of the respective compounds, thus maximizing the sensitivity of detection (see Supplementary Figures S6–S11).

For constructing calibration curves, TCA solutions in chloroform covering a range of concentrations, spiked with a known amount of MIR, were prepared and analyzed as described above. In total, 20 blanks containing only MIR were also injected in order to assess the limits of detection and quantification for each TCA [78]. For recovery testing from aqueous samples, 3.00 mL of solutions from each TCA in water having exact concentrations (1.00 and 20.0 μg/mL) were evaporated to dryness at 65 °C and re-taken up with chloroform and known amounts of MIR, then injected for GC-MS analysis under the same conditions. The same protocol was applied to solutions of each TCA with each of the two aforementioned concentrations plus 20.0 μg/mL of either BA, OA, MA or 35.0 μg/mL of CA. Recovery tests were performed in triplicate for each concentration of each TCA without and with each of the carboxylic acid.

### 3.4. General Photodegradation Procedure

In a typical photodegradation kinetics experiment, 14 aqueous samples of 3.00 mL of TCA of 20.0 μg/mL were pipetted into identical 7.00 mL quartz test tubes (100 mm in length; 13 mm in diameter) and capped. All TCA solutions with this concentration had a pH of 6. Considering that pKa values of TCAs range from a low of 8 (TRI) to a high of 9.4 (AMI) [79], the dominant molecular species in aqueous environment undergoing photodegradation was the protonated form for all TCAs. In cases where carboxylic acid additives were also used, adequate volumes of concentrated solutions of TCAs and concentrated aqueous solutions of carboxylic acids were mixed, then the mixtures were diluted to a final volume of 3.00 mL with water, such that both the final TCA and carboxylic acid concentrations were 20.0 μg/mL, with the exception of citric acid where 35.0 μg/mL was used due to its higher molar mass in order to ensure approximately the same molarity as the other additives. All solutions of TCA + carboxylic acid had a pH of 5, hence TCAs existed also in their protonated molecular form during these latter photodegradation experiments.

The test tubes were placed in a 14-slot rotating carousel, which was in turn placed in the center of the photoreactor, 8 cm away from the lamps on each side. The photoreactor, model LZC-4X (Luzchem Research Inc., Ottawa, ON, Canada), was equipped with eight identical lamps, four on the right side and four on the left side, and had a cooling system that maintained temperature at 25 ± 2 °C. Three different types of lamps, also from Luzchem Canada, were used, with emission maxima in the following domains: UVA (352 nm), UVB (313 nm), and UVC (254 nm). The total photon flux emitted by all eight lamps was 1576 μE m^−2^ s^−1^ for UVA lamps, 864 μE m^−2^ s^−1^ for UVB, and 1592 μE m^−2^ s^−1^ in the case of UVC lamps.

The quartz tubes were taken out of the photoreactor chamber one at a time, after various photodegradation periods, evaporated to dryness at 65 °C, redissolved together with a precisely known amount of internal standard in a total volume of chloroform equal to that of the evaporated water and analyzed chromatographically as described in Section 3.3. Every photodegradation experiment under a given set of conditions was replicated three times. Dark control experiments were also carried out in triplicate on both TCA alone and TCA + carboxylic acid aqueous solutions having the same concentrations as above using the same 14 quartz test tubes, rotating them in the carousel inside the photoreactor as described, but with the lamps kept off, and test tubes were removed and their solutions analyzed one at a time so as to cover a sufficient number of time-points spanning the whole range of photodegradation periods employed in all experiments.

### 3.5. Reuse of Citric Acid (CA) for Additional Photodegradation

In order to test the possibility of recovering and reusing the remaining CA after a TCA photodegradation round for an additional photodegradation round of a fresh batch of TCA, the following procedure was adopted. For each TCA, 14 quartz test tubes containing 20 μg/mL TCA + 35 μg/mL CA were subjected to UVC irradiation for two hours, i.e., a duration sufficient to ensure practically complete photodegradation of all TCAs in the presence of CA. After this time, the aqueous solution in every test tube was evaporated to dryness at 65 °C, then a fresh 3.00 mL portion of 20 μg/mL aqueous solution of the same TCA was added and stirred vigorously until all the CA leftover in the test tube from the previous photodegradation round was completely dissolved. Next, the photodegradation procedure described in Section 3.4 was followed for each TCA using the UVC lamps, with test tube sampling after various photodegradation periods and GC analysis of TCAs in order to determine their kinetics of photodegradation in the presence of recycled CA. This procedure was replicated three times for each TCA.

### 3.6. UV-Vis Spectra Measurement Procedure

UV-Vis spectra of TCAs in aqueous solution were measured using an Evolution 220 (Thermo Scientific Inc., Waltham, MA, USA) double-beam spectrophotometer, with a spectral range 190–1100 nm, using 3.5 mL quartz cuvettes with a 10 mm path length (ISOLAB Laborgeräte GmbH, Eschau, Germany) and doubly distilled water as blank. Stock aqueous solutions of TCAs were diluted to concentrations of 10 μg/mL (for IMI and CLO) and 5 μg/mL (for AMI, DOX, and TRI) and their UV-Vis spectra were recorded in the range 200–450 nm, where absorption bands were present. The two different dilution levels were chosen in order to achieve maximum absorbance values around 1 for all TCAs in this range.

## 4. Conclusions

In this study, an investigation of the comparative rates of photodegradation of five tricyclic antidepressant drugs (AMI, DOX, IMI, CLO, and TRI) in aqueous solution was performed using three different UV sources with emission maxima in the UVA, UVB, and UVC domains, respectively. The effect of adding carboxylic acids during photodegradation was also investigated. An internally validated GC-MS method was developed for the accurate quantification of antidepressants.

It was found that rates of photodegradation depend both on the type of UV irradiation source and the chemical structure of the drug. The fastest rates were attained with UVC and the slowest with UVA, while TCAs with a higher number of heteroatoms (DOX, CLO) photodegraded faster than the rest. Comparative rates of photodegradation of the five TCAs under the various UV sources also showed good correlations with the UV absorption bands in the spectra of these drugs recorded in aqueous medium. Under UVC light, for most TCAs the addition of carboxylic acids in the aqueous solution enhanced their photodegradation rates and thus reduced the irradiation time needed to completely degrade them. This effect was more pronounced for citric acid, followed by oxalic acid and then maleic acid, while benzoic acid was the least effective. In the case of citric acid, the possibility of reusing the remaining acid after one photodegradation round as an additive for one additional photodegradation round of a fresh batch of TCA was demonstrated and the additive showed comparable effectiveness.

The fundamental knowledge obtained through these results may serve multiple purposes. On the one hand, it offers information to pharmaceutical manufacturers and related stakeholders regarding the comparative photostability of these antidepressants when exposed to UV or even ordinary sunlight, known to include significant UVA and UVB components. Such information could guide their choice of packaging materials and storage conditions, or support more accurate estimates of drug shelf life. On the other hand, since antidepressant drug residues are a class of emerging contaminants of rising concern for municipal and industrial wastewaters, direct UV water treatment protocols may be developed in the future building upon these foundations. Future work should be geared toward up-scaling laboratory results to industrial installations, understanding in depth the mechanism by which carboxylic acids assist the photodegradation of tricyclic antidepressants, and exploring the feasibility of recycling and reusing other remaining carboxylic acids at the end of the photolysis procedure.

## Figures and Tables

**Figure 1:**
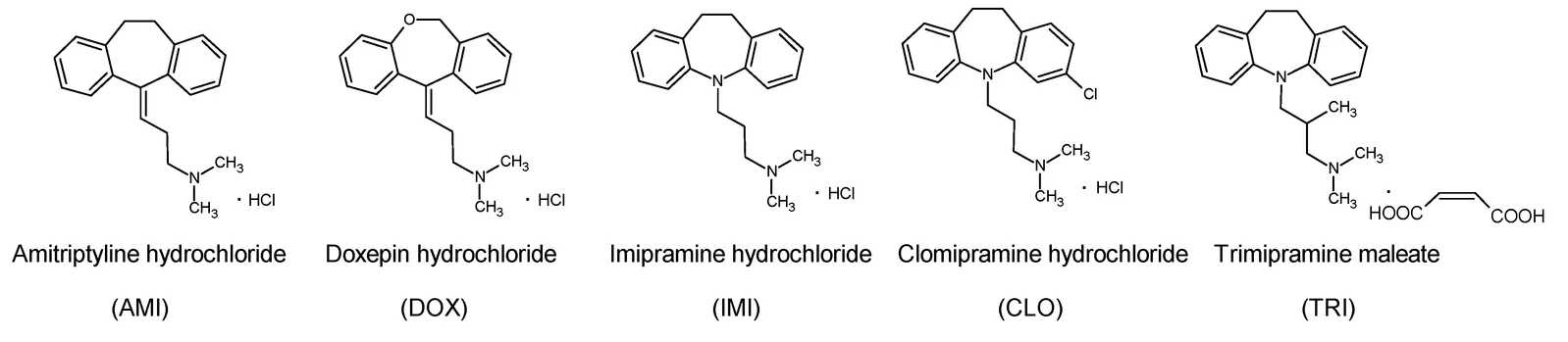
Chemical structures of the five tricyclic antidepressants (TCAs) investigated in this study (adapted from [7]).

**Figure 2:**
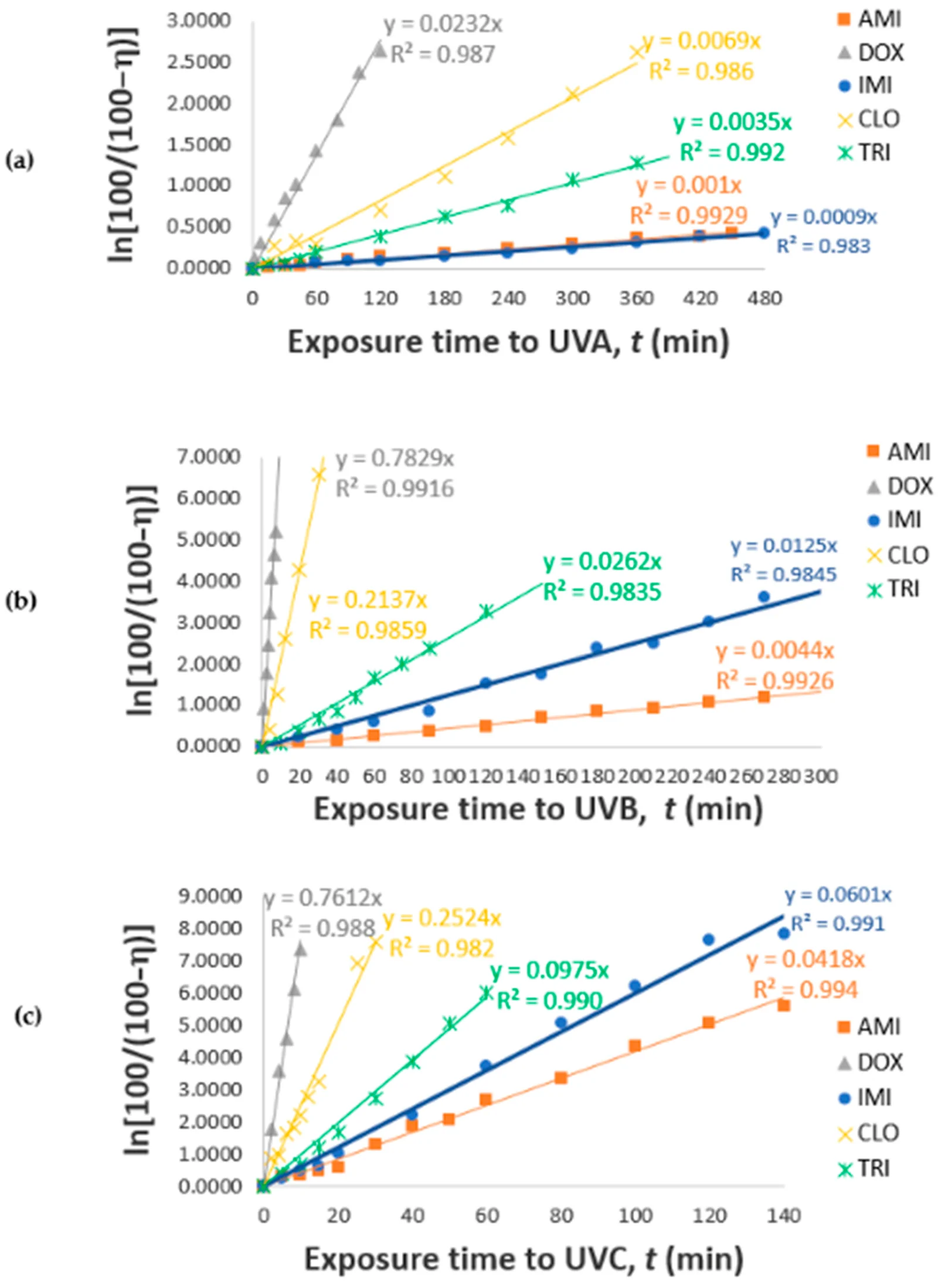
Kinetic curves of photodegradation processes of the five TCAs (AMI, DOX, IMI, CLO, and TRI) obtained upon exposure to UVA (**a**), UVB (**b**), and UVC (**c**) sources, respectively.

**Figure 3:**
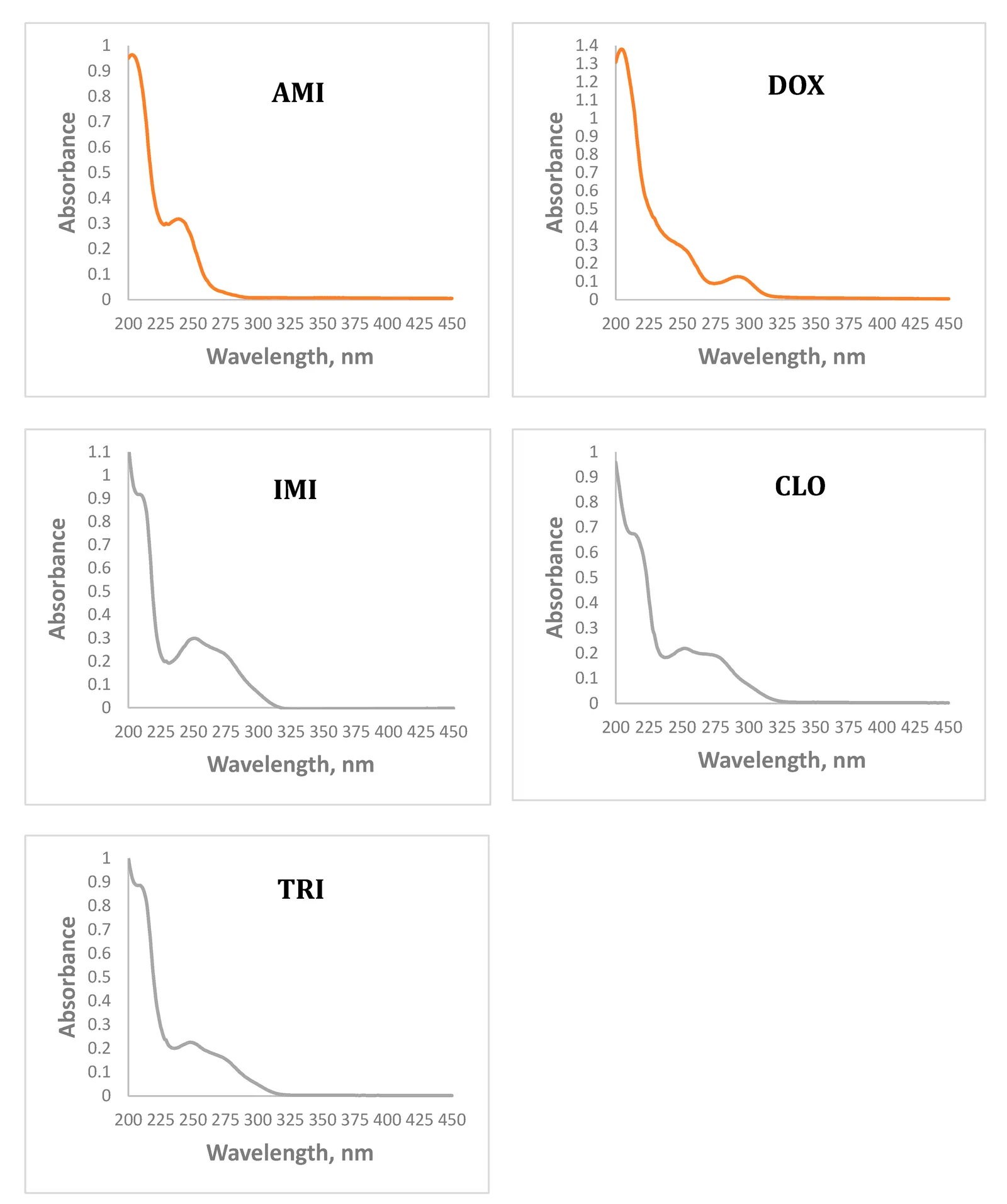
UV-Vis spectra of the five TCAs measured in aqueous solutions with concentrations 5 μg/mL (AMI, DOX, and TRI) and 10 μg/mL (IMI and CLO).

**Figure 4:**
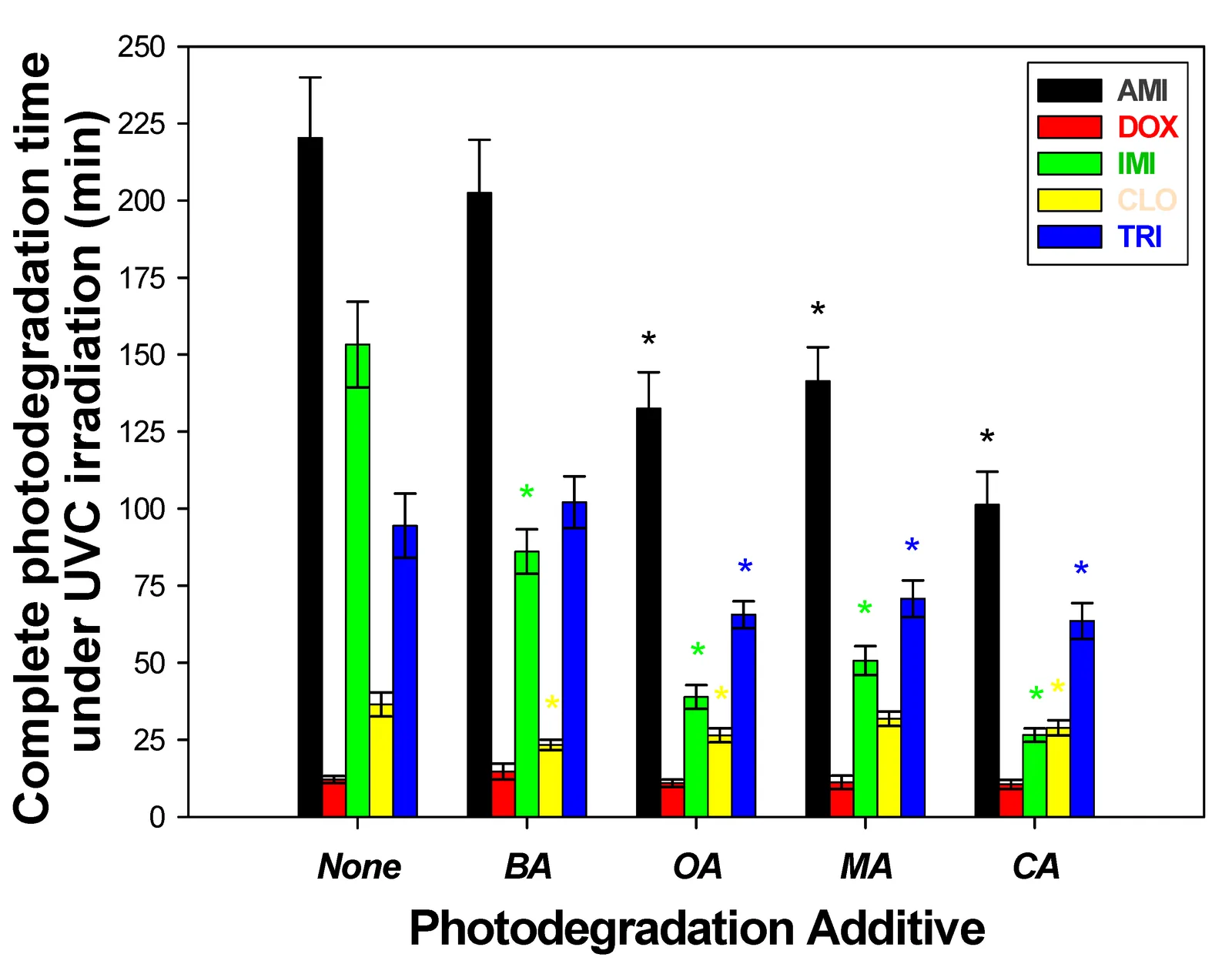
Comparison of complete photodegradation durations, *t*_∞_, under UVC irradiation of the five TCAs (AMI, DOX, IMI, CLO, and TRI) in aqueous solution without and with a photodegradation additive, namely: benzoic acid (BA), oxalic acid (OA), maleic acid (MA), or citric acid (CA). Error bars correspond to standard deviations in the mean (*n* = 3). Asterisks of one color indicate there is a significant difference between the time without additive and that with the respective additive in the case of the TCA corresponding to the same color (two-tailed *t* test, *p* < 0.05).

**Scheme 1:**
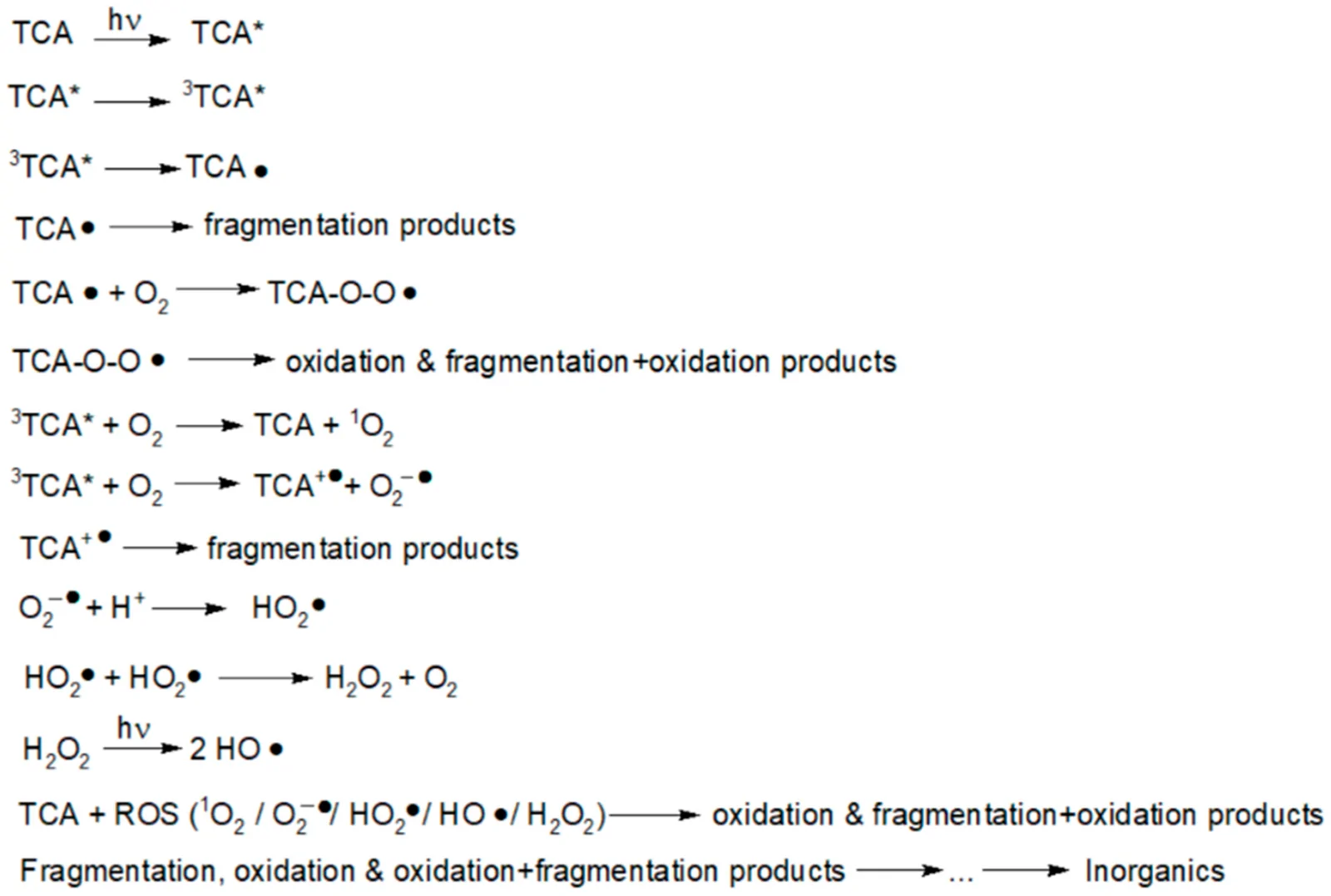
Proposed mechanism of UVC photodegradation of TCAs based on reaction intermediates reported in the literature.

**Table 1:** Figures of merit of the GC-MS method employed for quantification of TCAs from their aqueous solutions.

TCA	*m*/*z* Value	RT (min)	RRF	Linear Range (μg/mL)	R^2^	S (mL/μg)	LOD (ng/mL)	LOQ (ng/mL)	Recovery (%)		RSD_R_ (%)	
									1 μg/mL	20 μg/mL	1 μg/mL	20 μg/mL
AMI	58	17.2	0.245	10^−2^–10^2^	0.9948	3.62 × 10^5^	0.84	2.81	100.8	102.0	3.83	3.85
DOX	58	17.5	0.294	10^−2^–10^2^	0.9947	5.28 × 10^5^	3.78	12.61	97.2	98.9	2.04	2.29
IMI	234	17.4	0.521	10^−2^–10^2^	0.9999	4.96 × 10^5^	1.01	3.36	97.5	98.1	2.47	3.27
CLO	268	18.6	0.570	10^−2^–10^2^	0.9991	3.08 × 10^5^	4.76	15.86	98.4	97.5	3.79	2.16
TRI	249	17.3	1.451	10^−2^–10^2^	0.9998	1.28 × 10^6^	7.75	25.82	99.1	101.6	3.35	3.91

**Table 2:** Apparent pseudo-first-order rate constants, *k*_1_, and complete photodegradation durations, *t*_∞_, for the five TCAs (AMI, DOX, IMI, CLO, and TRI) in aqueous medium subjected to different UV sources. Values shown represent mean ± standard deviation (*n* = 3). Different capital letters mark significant differences in apparent rate constants between different UV sources for the same TCA and different lowercase letters mark significant differences between TCAs for the same UV source (two-tailed *t* test, *p* < 0.05).

	TCA:	AMI		DOX		IMI		CLO		TRI	
UV Source:		*k*_1_,	*t*_∞_,	*k*_1_,	*t*_∞_,	*k*_1_,	*t*_∞_,	*k*_1_,	*t*_∞_,	*k*_1_,	*t*_∞_,
		min^−1^	min	min^−1^	min	min^−1^	min	min^−1^	min	min^−1^	min
**UVA**		0.0010 ± 0.0002 ^Aa^	9210.3 ± 1842.1	0.0232 ± 0.0021 ^Ab^	397.0 ± 35.9	0.0009 ± 0.0001 ^Aa^	10233.7 ± 1137.1	0.0069 ± 0.0011 ^Ad^	1334.8 ± 212.8	0.0035 ± 0.0006 ^Ae^	2631.5 ± 451.1
**UVB**		0.0044 ± 0.0006 ^Ba^	2141.9 ± 285.4	0.7829 ± 0.0943 ^Bb^	11.8 ± 1.4	0.0125 ± 0.0008 ^Bc^	736.8 ± 47.2	0.2137 ± 0.0187 ^Bd^	43.1 ± 3.8	0.0262 ± 0.0034 ^Be^	351.5 ± 45.6
**UVC**		0.0418 ± 0.0049 ^Ca^	220.3 ± 25.8	0.7612 ± 0.0665 ^Bb^	12.1 ± 1.1	0.0601 ± 0.0072 ^Cc^	153.3 ± 18.4	0.2524 ± 0.0133 ^Cd^	36.5 ± 1.9	0.0975 ± 0.0114 ^Ce^	94.5 ± 11.0

**Table 3:** Apparent pseudo-first-order rate constants, *k*_1_, and complete photodegradation durations, *t*_∞_, for the five TCAs (AMI, DOX, IMI, CLO, and TRI) in aqueous medium subjected to UVC in the presence of fresh and recycled CA respectively. Values shown represent mean ± standard deviation (*n* = 3). Different capital letters mark significant differences in apparent rate constants between fresh and recycled CA in the case of the same TCA and different lowercase letters mark significant differences between TCAs for the same type of CA used (two-tailed *t* test, *p* < 0.05).

	TCA:	AMI		DOX		IMI		CLO		TRI	
UVC Light +		*k*_1_,	*t*_∞_,	*k*_1_,	*t*_∞_,	*k*_1_,	*t*_∞_,	*k*_1_,	*t*_∞_,	*k*_1_,	*t*_∞_,
		min^−1^	min	min^−1^	min	min^−1^	min	min^−1^	min	min^−1^	min
**Fresh CA**		0.0909 ± 0.0096 ^Aa^	101.3 ± 10.7	0.8772 ± 0.1253 ^Ab^	10.5 ± 1.5	0.3473 ± 0.0288 ^Ac^	26.5 ± 2.2	0.3186 ± 0.0268 ^Ac^	28.9 ± 2.4	0.1449 ± 0.0134 ^Ae^	63.6 ± 5.9
**Recycled CA**		0.0794 ± 0.0102 ^Aa^	116.0 ± 14.9	0.7514 ± 0.0682 ^Ab^	12.3 ± 1.1	0.2839 ± 0.0315 ^Ac^	32.4 ± 3.6	0.2670 ± 0.0284 ^Ac^	34.5 ± 3.7	0.1032 ± 0.0116 ^Ba^	89.2 ± 10.0

## Data Availability

The original contributions presented in this study are included in the article and Supplementary Materials. Further inquiries can be directed to the corresponding authors.

## Supplementary Materials

The following supporting information can be downloaded at https://www.mdpi.com/article/10.3390/ijms27208940/s1.

## Abbreviations

The following abbreviations are used in this manuscript:

AMI	Amitriptyline hydrochloride
AOP	Advanced oxidation process
BA	Benzoic acid
CA	Citric acid
CECs	Contaminants of emerging concern
CLO	Clomipramine hydrochloride
DOX	Doxepin hydrochloride
GC	Gas chromatography
GC-MS	Gas chromatography–mass spectrometry
IMI	Imipramine hydrochloride
IS	Internal standard
LOD	Limit of detection
LOQ	Limit of quantification
MA	Maleic acid
MIR	Mirtazapine
MS	Mass spectrum/spectrometry
OA	Oxalic acid
OECD	Organization for Economic Co-operation and Development
ROS	Reactive oxygen species
RRF	Relative response factor
RSD	Relative standard deviation
RT	Retention time
S	Sensitivity
SIM	Selected/single-ion monitoring
TCA	Tricyclic antidepressant
TRI	Trimipramine maleate
UV	Ultraviolet
UVA	Ultraviolet A
UVB	Ultraviolet B
UVC	Ultraviolet C
UV-Vis	Ultraviolet–visible
WHO	World Health Organization
