# Supplementary material for: Influence of UV Irradiation Source and of Carboxylic Acid Additives on the Kinetics of Photodegradation of Five Tricyclic Antidepressant Drugs in Aqueous Medium
Source: Int J Mol Sci. 2026 Oct 9;27(20):8940. doi: 10.3390/ijms27208940 (PMC13704724; doi:10.3390/ijms27208940)
Supplement: Supplementary file 1 [file ijms-27-08940-s001.zip › ijms-4549141-supplementary.pdf]

Supplementary Materials

Influence of UV Irradiation Source and of Carboxylic Acid Additives on the Kinetics of Photodegradation of Five Tricyclic Antidepressant Drugs in Aqueous Medium

Maria D. Ciuca and Radu C. Racovita *

National University of Science and Technology POLITEHNICA Bucharest, Faculty of Chemical Engineering and Biotechnologies, Department of Inorganic Chemistry, Physical Chemistry and Electrochemistry, 1-7 Gh. Polizu St., District 1, Bucharest 011061, Romania; maria980804@gmail.com

* Correspondence: radu.racovita@upb.ro

Table S1. Average recoveries and corresponding RSD_R values obtained from aqueous solutions containing both a TCA at either the 1 μg/mL or 20 μg/mL concentration level and a carboxylic acid at 20 μg/mL (BA, OA, MA) or 35 μg/mL (CA).

TCA	*Carboxylic acid additive*	*Recovery (%)*		*RSD_R (%)*	
		1 μg/mL	20 μg/mL	1 μg/mL	20 μg/mL
AMI	20 μg/mL BA	96.8	98.2	2.05	3.22
	20 μg/mL OA	98.9	100.5	2.84	3.03
	20 μg/mL MA	97.7	98.5	3.11	3.64
	35 μg/mL CA	101.6	96.9	4.15	3.63
DOX	20 μg/mL BA	96.2	98.8	2.24	2.49
	20 μg/mL OA	97.1	97.6	3.89	2.45
	20 μg/mL MA	96.4	99.3	3.06	4.01
	35 μg/mL CA	95.8	97.5	3.27	3.02
IMI	20 μg/mL BA	98.6	98.1	3.17	3.80
	20 μg/mL OA	99.2	98.3	2.19	3.45
	20 μg/mL MA	96.2	98.0	3.82	4.44
	35 μg/mL CA	100.8	98.8	4.25	4.38
CLO	20 μg/mL BA	95.3	98.6	3.53	4.16
	20 μg/mL OA	94.8	95.1	4.87	4.66
	20 μg/mL MA	98.1	97.4	4.12	3.71
	35 μg/mL CA	95.9	95.9	4.58	3.90

TRI	20 μg/mL BA	96.0	99.1	3.07	3.21
	20 μg/mL OA	98.5	101.3	4.13	4.69
	20 μg/mL MA	97.6	95.7	1.85	2.94
	35 μg/mL CA	96.4	96.9	2.64	3.37

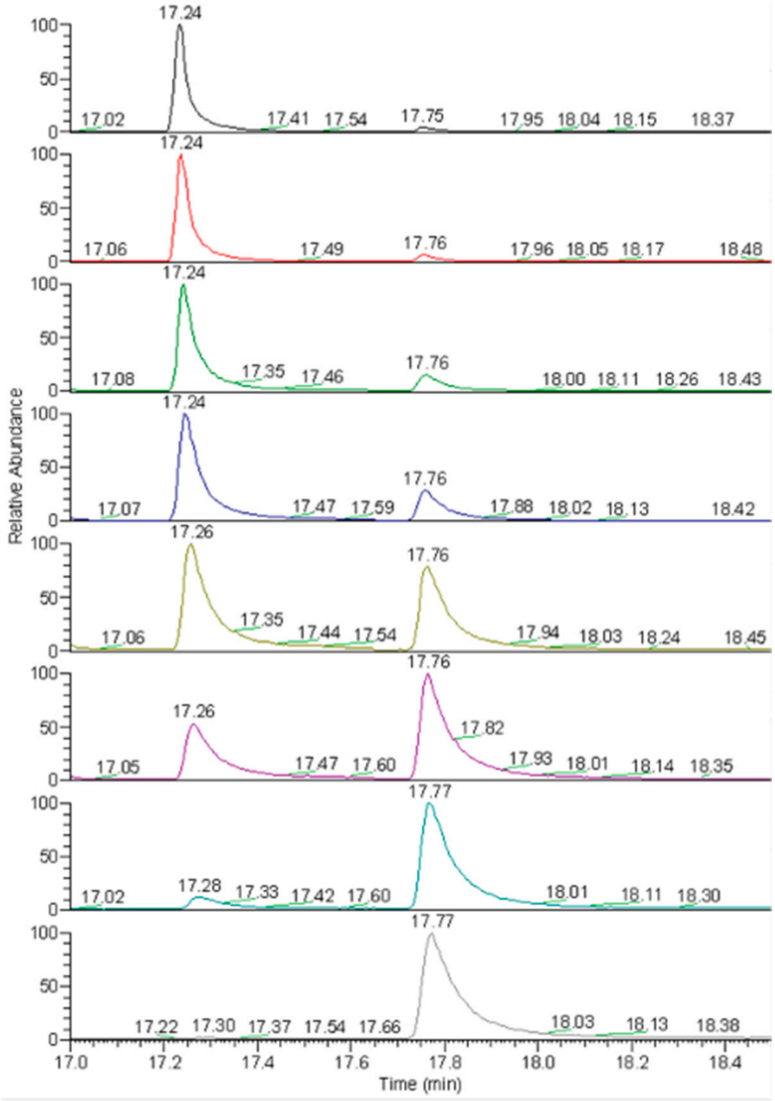

Figure S1. SIM chromatograms of AMI (RT~17.2 min), initially at 20 μg/mL, with increasing UVC photodegradation time (from top to bottom: 0, 10, 20, 30, 40, 50, 80, 140 min). The relative abundance of the IS (RT~17.8 min) peak area increases because the relative abundance of the AMI peak area decreases due to its photodegradation, but the total ion count of the IS peak is constant in all panels since all samples contain 1 μg/mL IS added just before injection into the GC.

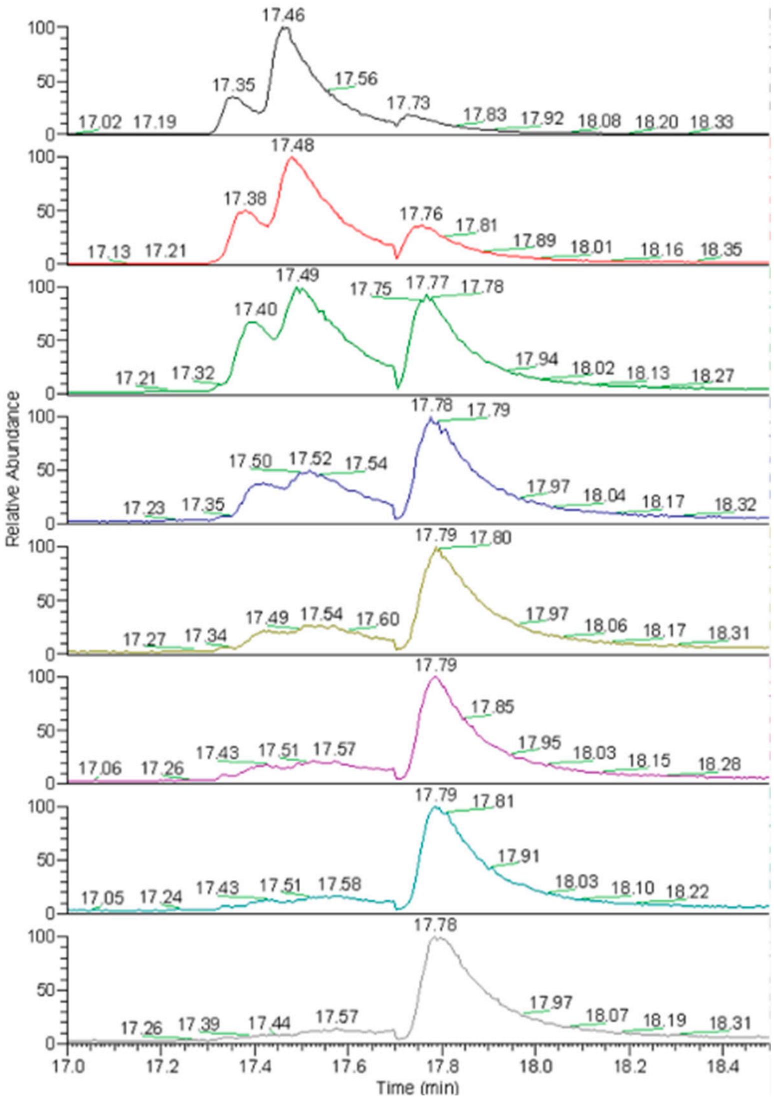

Figure S2. SIM chromatograms of DOX (mixture of 15% Z isomer with RT~17.4 min and 85% E isomer with RT~17.5 min), initially at 20 µg/mL, with increasing UVC photodegradation time (from top to bottom: 0, 2, 4, 5, 6, 7, 8, 10 min). The relative abundance of the IS (RT~17.8 min) peak area increases because the relative abundance of the cumulated DOX peak area decreases due to its photodegradation, but the total ion count of the IS peak is constant in all panels since all samples contain 1 µg/mL IS added just before injection into the GC.

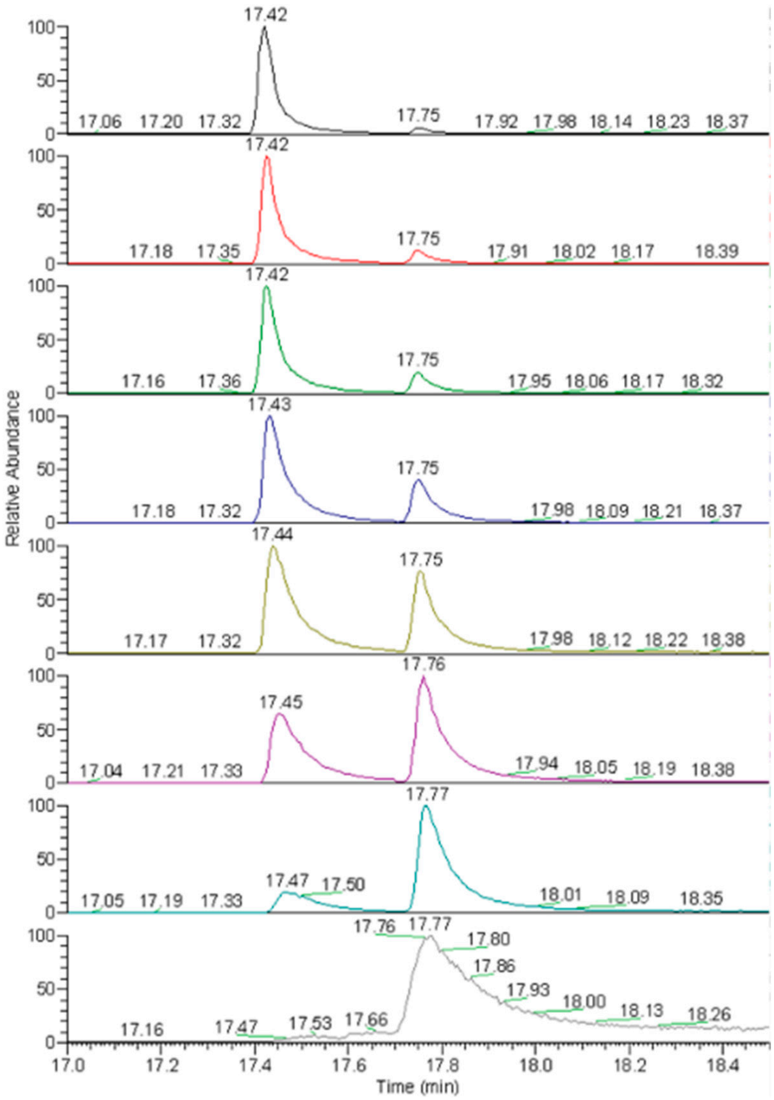

Figure S3. SIM chromatograms of IMI (RT~17.4 min), initially at 20 μg/mL, with increasing UVC photodegradation time (from top to bottom: 0, 5, 10, 20, 40, 60, 80, 120 min). The relative abundance of the IS (RT~17.8 min) peak area increases because the relative abundance of the IMI peak area decreases due to its photodegradation, but the total ion count of the IS peak is constant in all panels since all samples contain 1 μg/mL IS added just before injection into the GC.

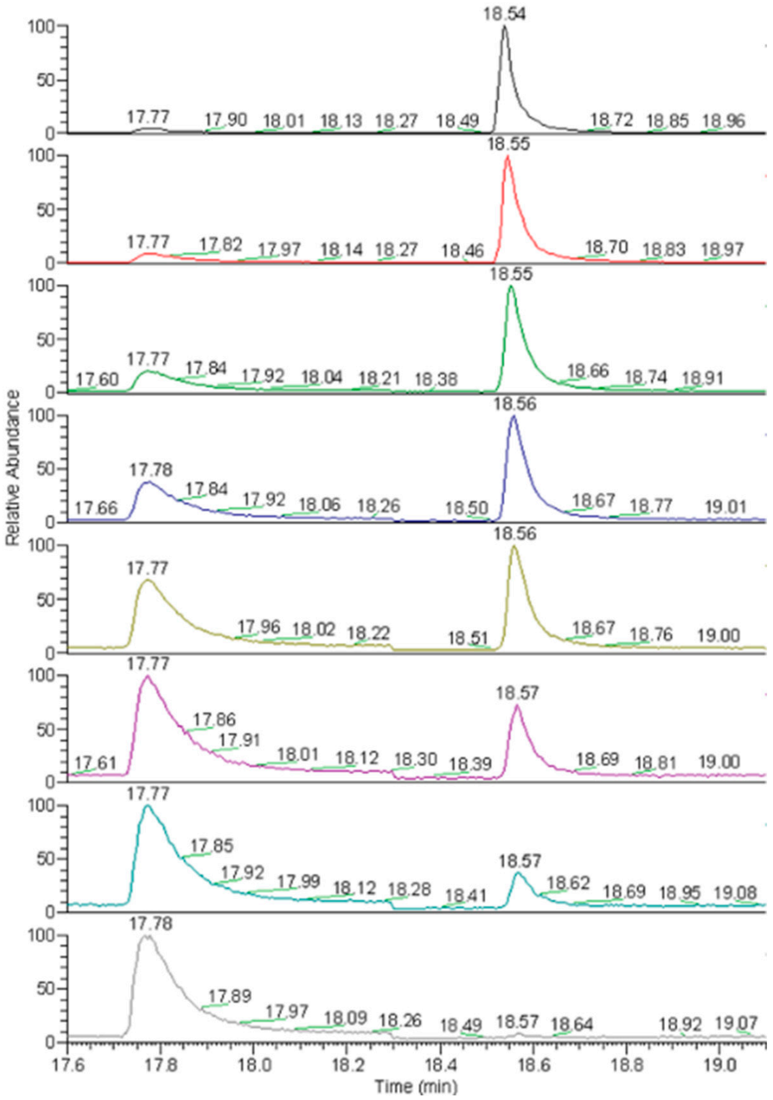

Figure S4. SIM chromatograms of CLO (RT~18.6 min), initially at 20 μg/mL, with increasing UVC photodegradation time (from top to bottom: 0, 2, 4, 8, 10, 15, 20, 25 min). The relative abundance of the IS (RT~17.8 min) peak area increases because the relative abundance of the CLO peak area decreases due to its photodegradation, but the total ion count of the IS peak is constant in all panels since all samples contain 1 μg/mL IS added just before injection into the GC.

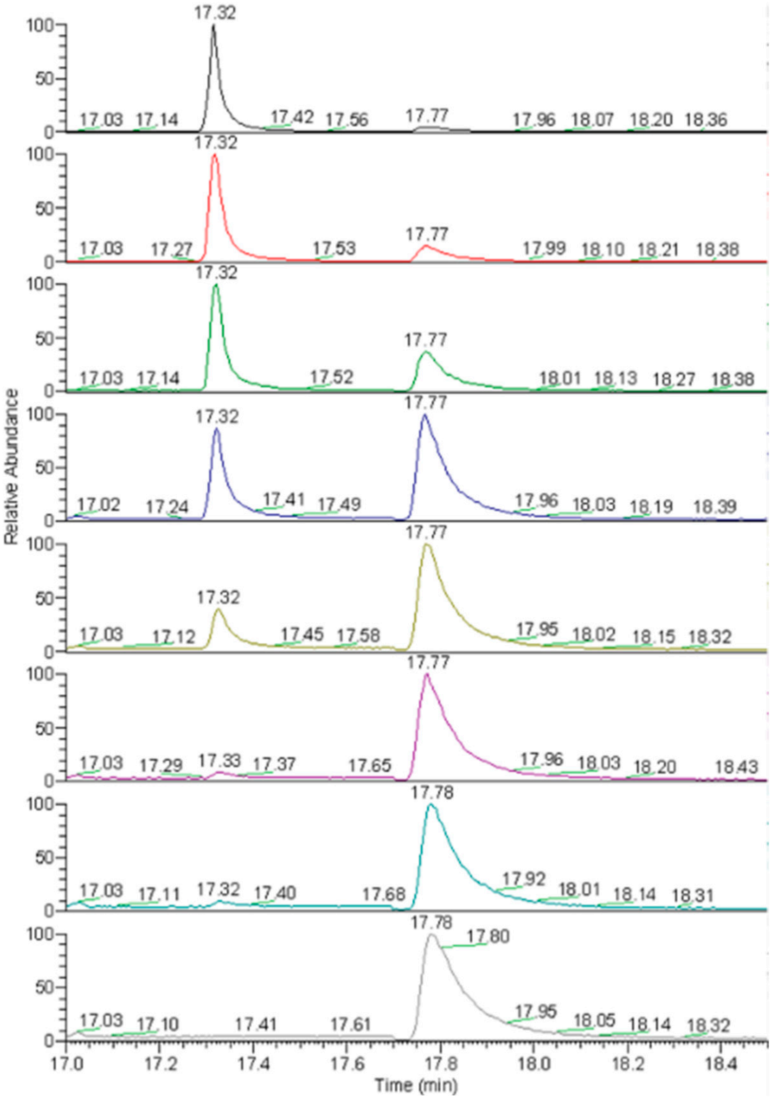

Figure S5. SIM chromatograms of TRI (RT~17.3 min), initially at 20 μg/mL, with increasing UVC photodegradation time (from top to bottom: 0, 10, 20, 30, 40, 50, 60, 70 min). The relative abundance of the IS (RT~17.8 min) peak area increases because the relative abundance of the TRI peak area decreases due to its photodegradation, but the total ion count of the IS peak is constant in all panels since all samples contain 1 μg/mL IS added just before injection into the GC.

Table S2. Average TCA concentrations measured in time during UVA, UVB, UVC exposure of each of the five TCAs in aqueous medium, as well as in absence of any light exposure either without or with a carboxylic acid additive co-dissolved in the solution.

TCA	*t (min)*	*C_{TCA} (μg/mL)* **UVA**	*C_{TCA} (μg/mL)* **UVB**	*C_{TCA} (μg/mL)* **UVC**	*C_{TCA} (μg/mL)* **No light**	*C_{TCA} (μg/mL)* **No light + 20 μg/mL BA**	*C_{TCA} (μg/mL)* **No light + 20 μg/mL OA**	*C_{TCA} (μg/mL)* **No light + 20 μg/mL MA**	*C_{TCA} (μg/mL)* **No light + 35 μg/mL CA**
**AMI**	0	20.0	20.0	20.0	20.0	20.0	20.0	20.0	20.0
	5			15.2					
	10			14.2					
	15	20.0		12.4					
	20		18.1	11.1	19.9	19.8	20.1	19.8	19.9
	30	19.5		5.4					
	40		17.4	3.2	20.1	20.2	20.1	19.9	20.0
	45	19.3							
	50			2.5					
	60	18.6	15.4	1.4	19.9	20.2	19.8	19.6	19.9
	80			0.7					
	90	17.9	13.8		19.8	19.9	20.0	19.7	19.8
	100			0.3					
	120	17.5	12.6	0.1	20.0	20.1	20.3	19.8	19.8
	140			0.1					
	150		10.0		19.8	19.7	19.9	20.1	19.6
	180	16.6	8.5		19.9	19.9	19.8	19.9	19.6
	210		7.8		20.0	20.3	19.5	19.8	19.9
	240	15.7	6.8		19.7	19.8	19.7	19.6	20.1
	270		6.2						
	300	14.9			20.1	19.8	19.6	19.8	19.8
	360	13.8			19.8	19.7	19.7	19.5	20.0
	420	13.5			20.0	20.4	19.9	19.6	19.3
	450	13.0			19.8	19.9	19.6	19.8	19.5
**DOX**	0	20.0	20.0	20.0	20.0	20.0	20.0	20.0	20.0
	1		8.0	9.3	19.8	19.8	19.7	19.6	19.7
	2	17.7	3.3	3.4	19.6	19.8	19.8	19.7	19.5
	3		1.7	2.1	19.8	19.7	19.7	20.0	19.6
	4		0.8	0.6	19.7	19.7	19.6	20.1	19.9
	5		0.3	0.4	19.9	19.8	19.5	19.8	19.5
	6		0.2	0.2					
	7		0.1	0.1					
	8	14.6	0.1	0.0	19.6	19.6	19.8	19.7	19.4
	10		0.1	0.0	19.8	20.2	19.6	19.6	19.3
	20	11.1			19.8	19.7	20.1	20.0	19.5
	30	8.6			19.7	19.9	19.9	19.5	19.6
	40	7.2							
	60	4.8			19.6	19.6	19.8	19.9	19.3
	80	3.3							
	100	1.9			19.7	19.8	19.6	19.8	19.4
	120	1.4			19.4	19.7	19.6	19.6	19.2
	180				19.4	19.6	19.8	19.7	19.7

IMI	0	20.0	20.0	20.0	20.0	20.0	20.0	20.0	20.0
	5			15.6					
	10			12.5	20.2	19.9	20.2	19.7	20.0
	15			10.5					
	20		15.9	7.2	19.7	19.7	20.2	19.8	19.6
	30	19.5							
	40		13.6	2.2	19.9	20.3	19.8	19.8	20.2
	60	18.7	11.2	0.5	19.7	20.0	19.6	19.6	19.8
	80			0.1					
	90	18.2	8.6		19.7	19.9	19.5	19.8	19.5
	100			0.0					
	120	18.4	4.4	0.0	20.0	19.9	19.4	19.7	19.5
	140			0.0					
	150		3.5		20.1	20.1	19.7	19.2	19.6
	180	17.3	1.9		19.8	19.6	19.6	19.7	19.3
	210		1.7						
	240	16.8	1.0		19.7	19.7	19.4	19.5	19.6
	270		0.5						
	300	15.7			19.7	19.8	19.6	19.6	19.1
	360	14.7			19.8	19.8	19.8	19.4	19.5
	420	13.6			19.4	19.7	19.7	19.2	19.3
	480	13.0			19.6	19.8	19.3	19.6	18.8
CLO	0	20.0	20.0	20.0	20.0	20.0	20.0	20.0	20.0
	2			8.3					
	4		13.3	7.7	20.0	19.8	19.7	20.1	19.7
	6			3.9					
	8		5.8	3.2	19.6	19.6	19.7	20.2	19.5
	10			2.3					
	12		1.5	1.2	19.8	19.6	19.4	19.6	20.0
	15			0.8	19.7	20.2	20.0	19.8	19.7
	20	15.3	0.3	0.1	19.9	20.1	19.6	19.6	19.8
	25			0.0					
	30		0.0	0.0	19.6	19.7	19.7	19.6	19.8
	40	14.3							
	45		0.0		20.3	20.0	19.8	19.8	19.8
	60	14.9			19.6	19.9	19.9	19.7	19.5
	120	9.9			19.8	19.7	20.0	19.6	19.7
	180	9.0			19.8	20.1	19.5	19.8	19.4
	240	6.6			19.5	19.9	19.7	19.7	19.7
	300	4.1			19.7	19.6	19.8	19.6	19.7
	360	2.4			19.4	19.6	19.3	19.8	19.8
TRI	0	20.0	20.0	20.0	20.0	20.0	20.0	20.0	20.0
	5			13.6					
	10		18.8	10.3	19.8	20.3	19.7	19.8	19.6
	15	19.2		6.0					
	20		14.0	3.8	19.7	19.8	19.7	19.6	19.8
	30	19.1	10.3	1.3	19.9	19.6	19.5	19.9	20.0
	40		8.6	0.4	20.1	20.1	19.7	19.8	19.6
	45	17.9							
	50		6.2	0.1	19.7	19.9	19.9	19.6	19.7

	60	16.3	3.7	0.1	20.0	19.6	19.6	19.5	20.1
	70			0.0					
	75		2.7		19.8	19.7	19.7	19.8	19.8
	90		1.8		19.6	19.8	19.6	20.1	19.7
	120	13.6	0.8		19.9	19.9	19.6	20.2	19.7
	180	10.7			19.4	20.1	19.5	19.9	19.5
	240	9.4			19.6	19.6	19.8	19.7	19.2
	300	6.9			19.4	19.5	19.8	19.5	18.9
	360	5.5			19.7	19.5	19.4	19.7	18.7

Table S3. Chemical structures of some organic UV photodegradation products reported in the literature as intermediates en route to the complete mineralization of the five TCAs studied in this work.

TCA parent structure	*Product category*	*Chemical structures*	**Ref.**
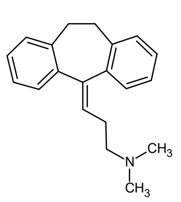	*Fragmentation products*	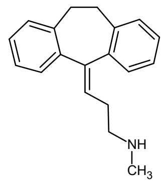 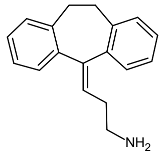	[33,57]
	Oxidation products	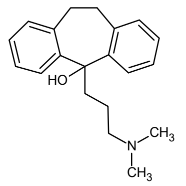 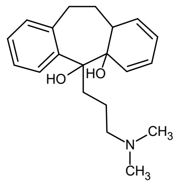 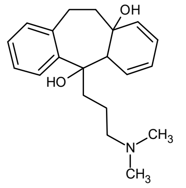	[22]
		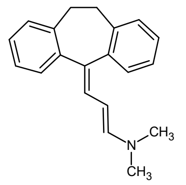 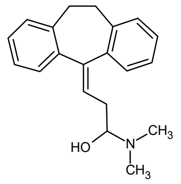 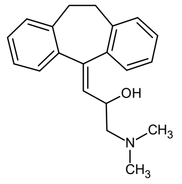	[33]
		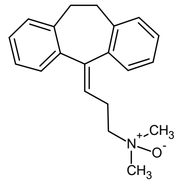 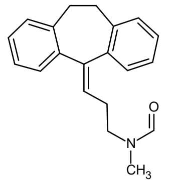	[57]
	Oxidation+fragmentation products	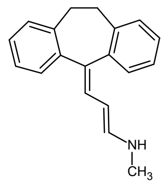 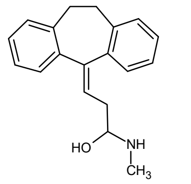 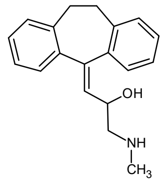	[33]
		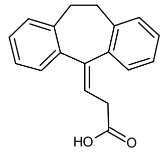 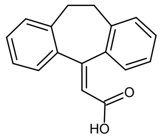	[57]
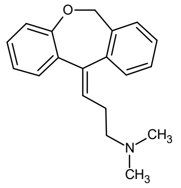	*Isomerisation products*	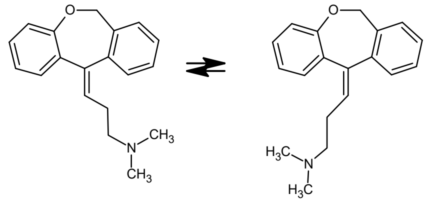	[29]
	Oxidation products	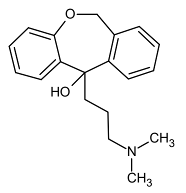 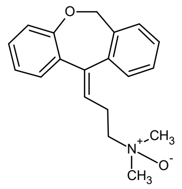	[54]

		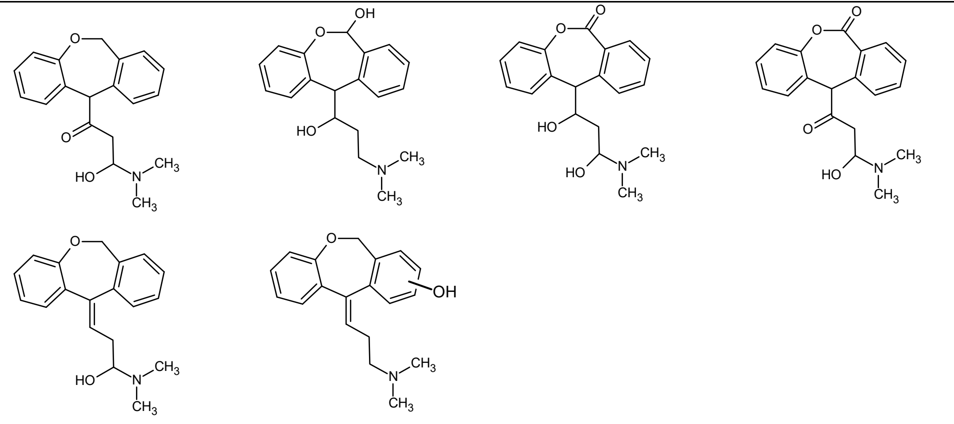	[25]
	Oxidation+fragmentation products	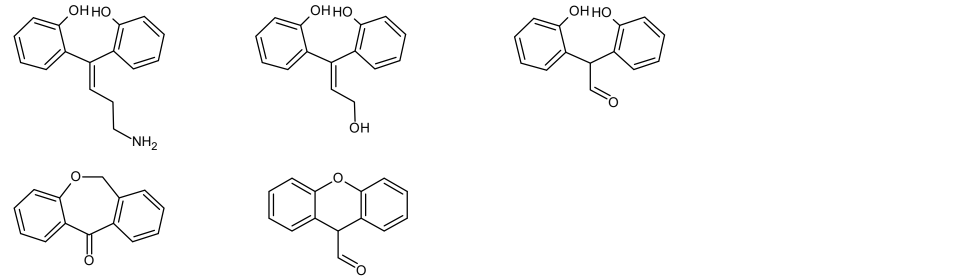	[25]
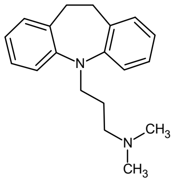	*Fragmentation products*	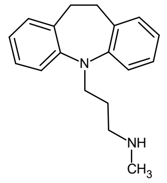 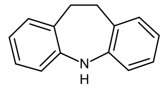	[55,56] [55]
	Oxidation products	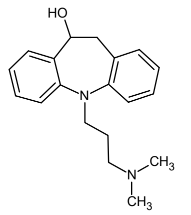 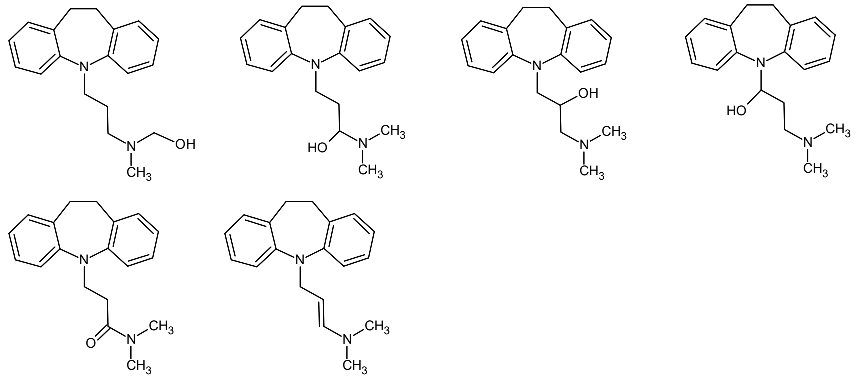	[55] [56]
	Oxidation+fragmentation products	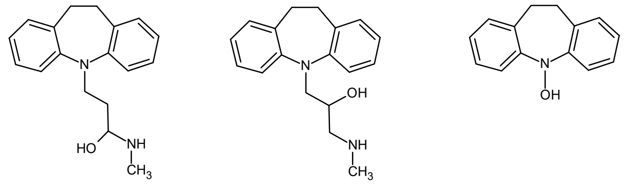	[56]

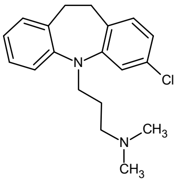	*Fragmentation products*	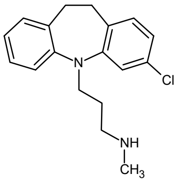	[54,57]
		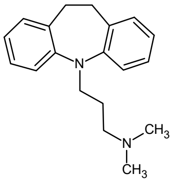	[54]
	Oxidation products	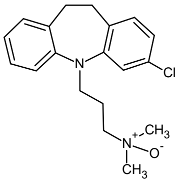	[57]
	Oxidation+fragmentation products	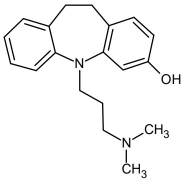	[22,54]
		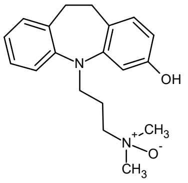	[54]
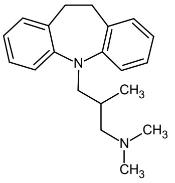	*Fragmentation products*	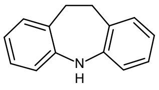 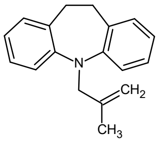	[23]
	Oxidation products	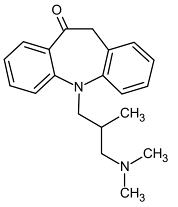 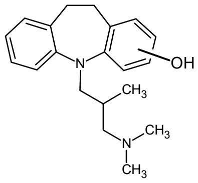 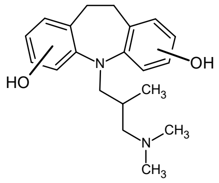	[23]
	Oxidation+fragmentation products	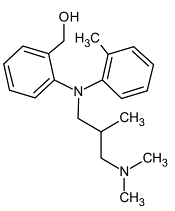 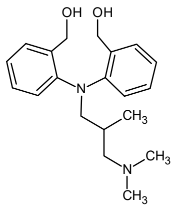 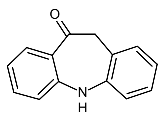 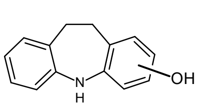 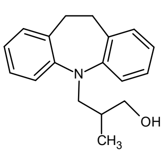 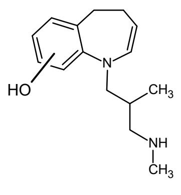 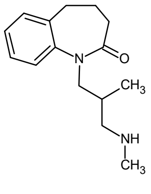	[23]

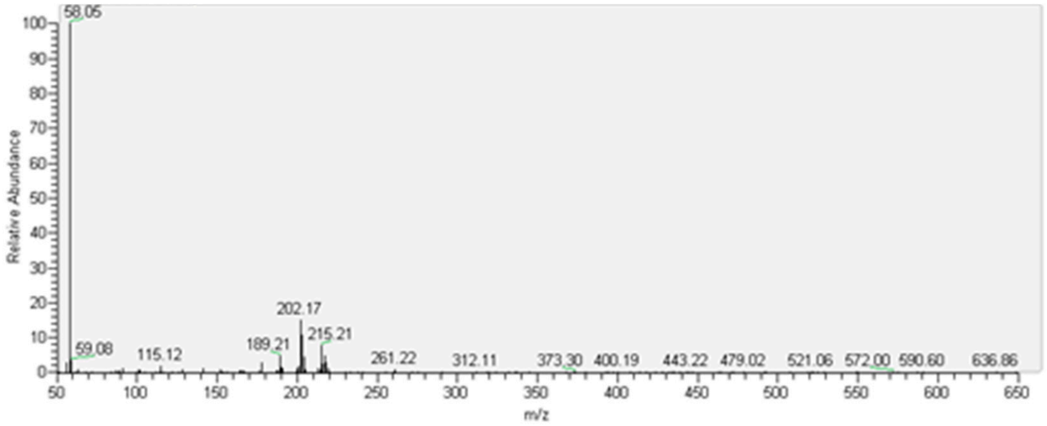

Figure S6. Electron ionization mass spectrum of amitriptyline (AMI).

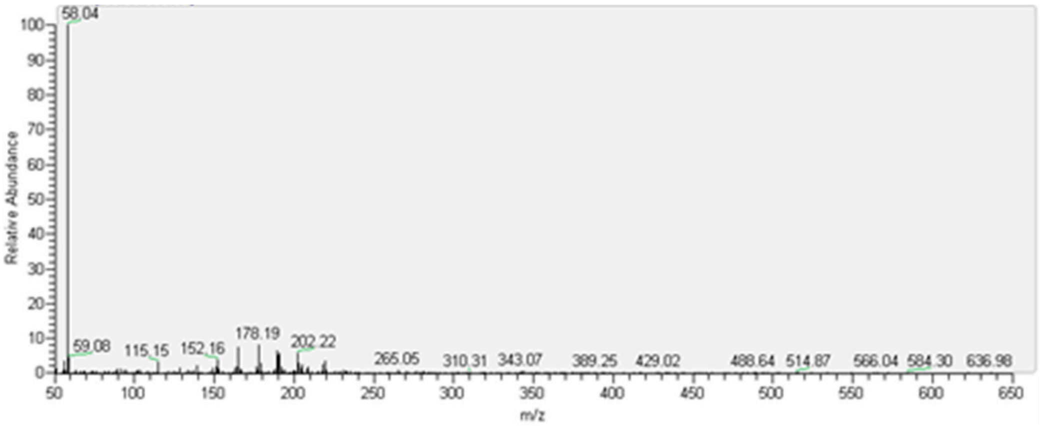

Figure S7. Electron ionization mass spectrum of doxepin (DOX).

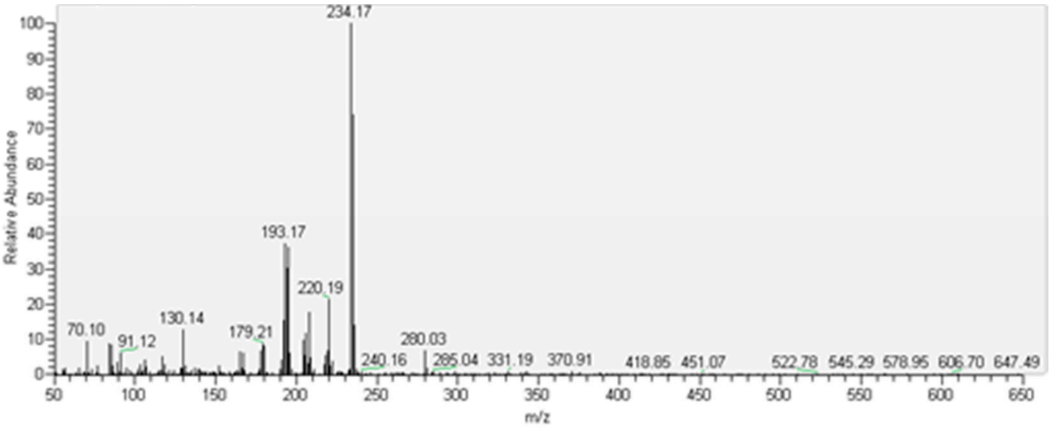

Figure S8. Electron ionization mass spectrum of imipramine (IMI).

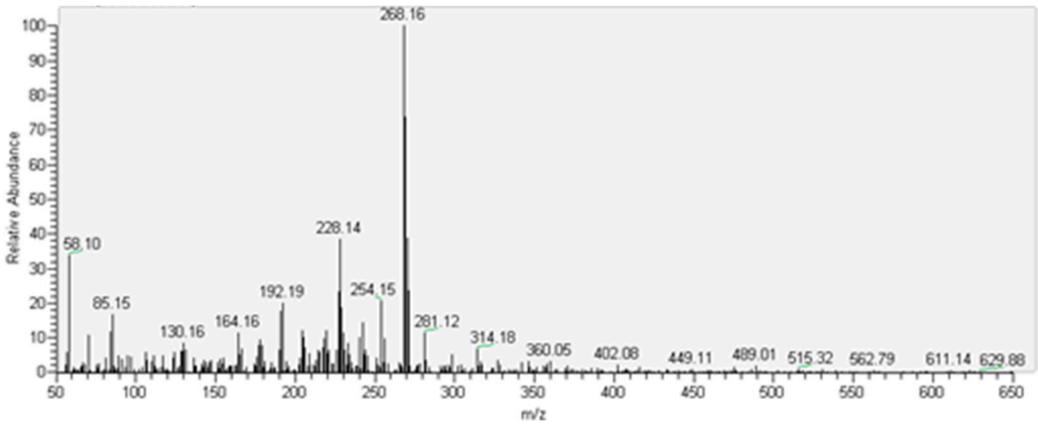

Figure S9. Electron ionization mass spectrum of clomipramine (CLO).

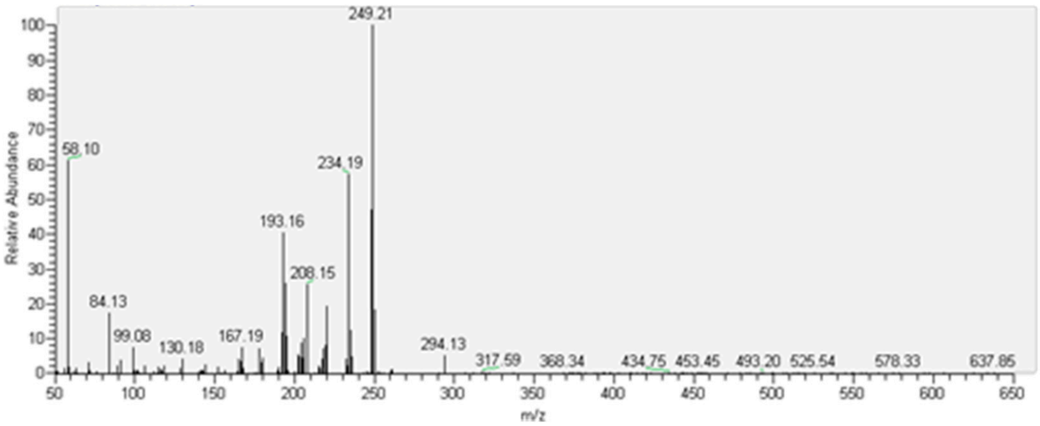

Figure S10. Electron ionization mass spectrum of trimipramine (TRI).

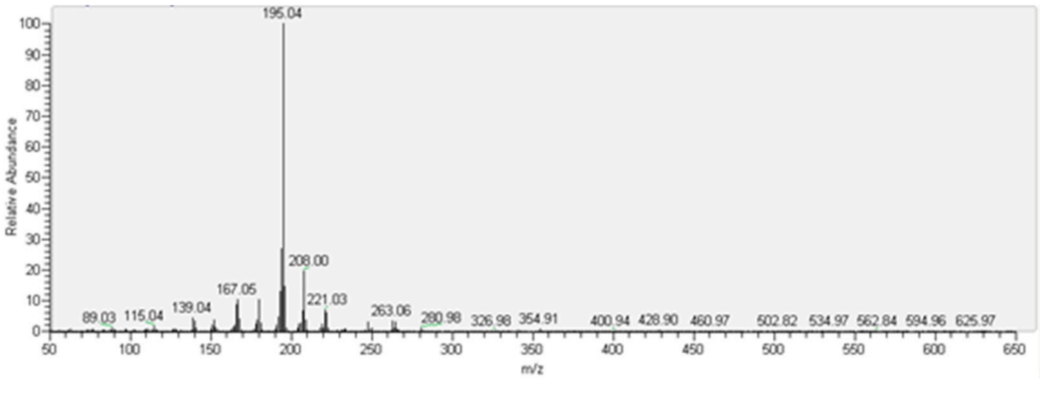

Figure S11. Electron ionization mass spectrum of mirtazapine (MIR).
